# Characterization of immunoglobulin *loci* in the gigantic genome of *Ambystoma mexicanum*

**DOI:** 10.3389/fimmu.2023.1039274

**Published:** 2023-01-27

**Authors:** Jesús Martinez-Barnetche, Elizabeth Ernestina Godoy-Lozano, Stephanie Saint Remy-Hernández, Diana Laura Pacheco-Olvera, Juan Téllez-Sosa, Humberto Valdovinos-Torres, Rodolfo Pastelin-Palacios, Horacio Mena, Luis Zambrano, Constantino López-Macías

**Affiliations:** ^1^ Centro de Investigación Sobre Enfermedades Infecciosas, Instituto Nacional de Salud Pública, Cuernavaca, Morelos, Mexico; ^2^ Escuela Nacional de Ciencias Biológicas, Instituto Politécnico Nacional, México City, Mexico; ^3^ Unidad de Investigación Médica en Inmunoquímica, UMAE Hospital de Especialidades, Centro Médico Nacional Siglo XXI, Instituto Mexicano del Seguro Social, México City, Mexico; ^4^ Facultad de Química, Universidad Nacional Autónoma de México, México City, Mexico; ^5^ Laboratorio de Restauración Ecológica, Instituto de Biología. Universidad Nacional Autónoma de México, México City, Mexico

**Keywords:** Ambystoma, antibodies, gigantic genome, pseudogenes, amphibia

## Abstract

**Background:**

The axolotl, *Ambystoma mexicanum* is a unique biological model for complete tissue regeneration. Is a neotenic endangered species and is highly susceptible to environmental stress, including infectious disease. In contrast to other amphibians, the axolotl is particularly vulnerable to certain viral infections. Like other salamanders, the axolotl genome is one of the largest (32 Gb) and the impact of genome size on Ig *loci* architecture is unknown. To better understand the immune response in axolotl, we aimed to characterize the immunoglobulin *loci* of *A. mexicanum* and compare it with other model vertebrates.

**Methods:**

The most recently published genome sequence of *A. mexicanum* (V6) was used for alignment-based annotation and manual curation using previously described axolotl Ig sequences or reference sequences from other vertebrates. Gene models were further curated using *A. mexicanum* spleen RNA-seq data. Human, *Xenopus tropicalis*, *Danio rerio* (zebrafish), and eight tetrapod reference genomes were used for comparison.

**Results:**

Canonical *A. mexicanum* heavy chain (IGH), lambda (IGL), sigma (IGS), and the putative surrogate light chain (SLC) *loci* were identified. No kappa *locus* was found. More than half of the IGHV genes and the IGHF gene are pseudogenes and there is no clan I IGHV genes. Although the IGH *locus* size is proportional to genome size, we found local size restriction in the IGHM gene and the V gene intergenic distances. In addition, there were V genes with abnormally large V-intron sizes, which correlated with loss of gene functionality.

**Conclusion:**

The *A. mexicanum* immunoglobulin *loci* share the same general genome architecture as most studied tetrapods. Consistent with its large genome, Ig *loci* are larger; however, local size restrictions indicate evolutionary constraints likely to be imposed by high transcriptional demand of certain Ig genes, as well as the V(D)J recombination over very long genomic distance ranges. The *A. mexicanum* has undergone an extensive process of Ig gene loss which partially explains a reduced potential repertoire diversity that may contribute to its impaired antibody response.

## 1 Introduction

Adaptive immunity is a vital feature of the vertebrate immune system. It relies on generating a vast antigen receptor repertoire clonally distributed on the surface of B and T cells. Antigen receptor repertoire is generated independently of antigen by somatic recombining V, D, and J segments in primary lymphoid organs. In the periphery, antigen clonally selects B and T cells bearing high-affinity antigen receptors for the selecting antigen. Further antigen-dependent diversification is achieved by somatic hypermutation (SHM) and class switch recombination (CSR).

Despite the mechanistic and structural commonalities involved in the generation of vertebrate adaptive receptor repertoire diversity, there is considerable evolutionary plasticity of Ig *loci* that have originated lineage-specific variation, manifested as alternative and diversified structures with novel recognition and functional capabilities. According to the birth and death model, Ig *loci* evolve by gene duplication, diversification, and pseudogenization that explain lineage differences in the type and number of functional antibody classes and V, D, and J segments (1–3).

*Ambystoma mexicanum* is a unique caudate amphibian endemic of central Mexico and is currently an endangered species. It is a neotenic organism capable of complete limb and nervous system regeneration (4). Like many amphibians, increased susceptibility to certain infectious diseases may play a role in the axolotl population decrease (5, 6).

Most of our amphibian adaptive immune system knowledge derives from the anurans *Xenopus leavis* and *X. tropicalis*, metamorphosing frogs (7) in which antibodies are encoded by a translocon-type IGH *locus* that includes IgM, IgD, IgX, IgY, and IgF heavy chain classes, and three light chain *loci*: λ, κ and σ (3, 8, 9). The divergence between anurans and caudates is estimated to occur 292 my ago (10). The antibody immune response is crucial for controlling or preventing viral infections (11, 12). *Ambystoma* sp., but not other salamanders and not anuran amphibians are particularly vulnerable to certain viral infections (13–16). To gain further knowledge to make generalizations about the amphibian adaptive immune system, a thorough characterization of non-anuran species is required.

*A. mexicanum* genome has been sequenced. It is 10 times larger than the human genome (32 Gigabases) and 17 times larger than *X. tropicalis* (17). Further efforts involving SNP´s segregant mapping (18) and Hi-C allowed a 14 chromosome-level assembly (19). The genome analysis shows that LTR retrotransposons have contributed to genome size increase in the axolotl. Moreover, lower rates of small DNA deletion compared to other tetrapods may contribute to the large genome in salamanders (20).

V(D)J recombination is a complex mechanism that depends on non-coding transcription, deep chromatin reorganization, double-stranded DNA breaks and repair, over long DNA intervals within Ig and TCR *loci* to proceed successfully, while avoiding genomic instability (21). Whether there is a length limit in antigen receptor *loci* for an efficient V(D)J recombination remains an open question.

To better understand the immune response in non-anuran ectotherms and explore the impact of increased genome size in adaptive antigen receptor *loci*, we report a comprehensive structural map, annotation, and functional characterization of the heavy and light chains immunoglobulin *loci* in axolotl. Moreover, we provide evidence that the IGHM gene and the IGHV and IGLV cluster size have not grown proportionally to the genome size.

## 2 Results

### 2.1 *A. mexicanum* heavy chain *locus* (IGH)

**Figure 1:**
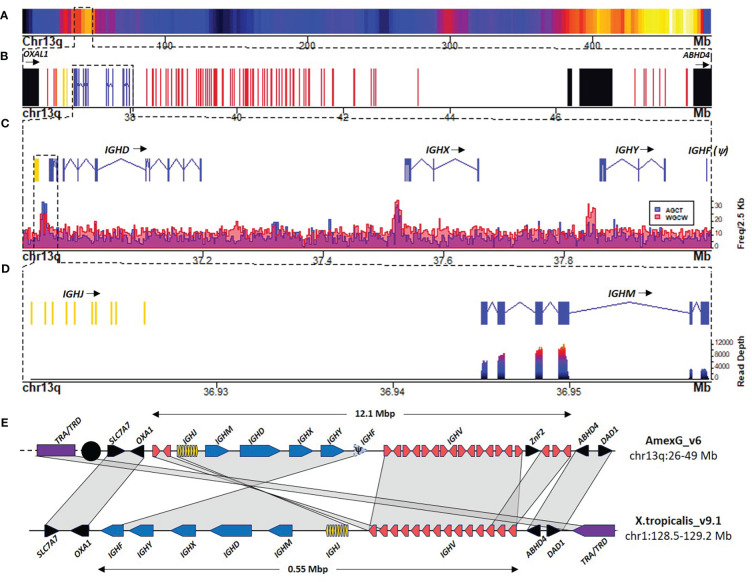
The heavy chain *locus* is located in the centromeric portion of chr13q (483 Mbp). **(A)** Gene density plot of chr13q where the IGH *locus* is encoded (box). Dark blue colors indicate low gene density. **(B)** Zoom of the whole IGH *locus* (35.967 – 48.91 Mbp). Non-Ig genes (black) in proximal flank *OXA1L* gene and distal flank *ABHD4*, IGHC genes (blue), IGHV genes (red), and IGHD and IGHJ (yellow). **(C)** Zoomed view (36.9 – 38.09 Mbp) of the IGHJ-IGHC gene cluster. The lower panel shows AID hotspots density per 2.5 Kb along the IGHJ-IGHC cluster to map Switch regions. **(D)** Close-up of the IGHJ cluster and the IGHM gene. The bottom panel shows the spleen RNA-seq coverage histogram of the IGHJ-IGHM region. **(E)** Schematic representation of IGH *locus* in *A. mexicanum* genome (v6) and the corresponding *locus* in *X. tropicalis*. Color code as in A *X. tropicalis* displays the canonical architecture, in which the V, J and C clusters have the same orientation. Note that in *A. mexicanum* the JH-CH cluster and 3 IGHV segments appear to be inverted, which seems implausible due to the mechanism of V(D)J recombination. Moreover, IGHV segment orientation in axolotl are intercalated, which is atypical. Interestingly, in axolotl, the TRA/TRD *locus* (Yellow) is not linked to the IGH *locus* as in *X. tropicalis* and is coded across the centromere (black circle) in chr13p: 3.3-7.7 Mbp). Not on scale. The IGHD cluster is not shown for simplicity.

We identified the IGH *locus* at 36.4 - 48.5 Mbp of chromosome 13q. The whole *locus* spans 12.1 Mbp and, as in *X. tropicalis*, is flanked by the *SCL7A7* and *OXAL1* genes in the centromeric flank and the *ABDH4* and *DAD1* genes in the telomeric flank (**Figures 1A, B, E**). We confirmed the observations suggesting that in *X. tropicalis*, the IGH *locus* is genetically linked to the TCRα/TCRδ *locus* (22). Indeed, *X. tropicalis* IGH was mapped to chromosome 1 (128.3 - 128.9 Mbp) and is only separated from the TCRα/TCRδ *locus* (128.95 - 129.5 Mbp) by the *ABDH4* and *DAD1* genes (**Figure 1E**). A detailed annotation file is provided in **Supplementary File 2**; **Table S1**. In contrast, the *A. mexicanum* IGH *locus* is delinked from the TCRα/TCRδ *locus*, which is found across the centromere in chr13p (3.5 - 6.5 Mbp) (Manuscript in preparation).

The usual organization of tetrapod IG and TCR *loci* is in translocon, with a cluster of IGHV genes (upstream), followed by the (D) IGHJ, and IGHC genes (downstream), all in the same transcriptional orientation. The *A. mexicanum* IGH *locus* displays an unusual organization: it starts with a 3 IGHV segment subcluster, followed by the IGHD, IGHJ, and the IGHC clusters in the same orientation (from centromere to telomere). However, downstream of the IGHC cluster is a large cluster of 85 IGHV genes delimited by the *ABDH4* and *DAD1* genes (**Figure 1B**). Moreover, the orientation of IGHV segments of both clusters is irregularly intercalated, with three large stretches of 19, 12, and 10 IGHV genes in the same direction, intercalated with small stretches of IGHV in the opposite transcriptional orientation (**Supplementary File 1**; **Figure S1**). These anomalies are likely derived from assembly errors. We propose an IGH *locus* model based on synteny with *X. tropicalis*, in which the first IGHV cluster, the IGHD, IGHJ, and IGHC conform to a single DNA block in inverted orientation so that it follows the canonical V(D)JC order in the same transcriptional orientation (**Figure 1E**).

### 2.2 IGHC genes

The *A. mexicanum* IGHC cluster is 1.1 Mbp long (Chr13q: 36.94 - 38.03 Mbp), and its structure is very similar to *X. tropicalis* (8) IGHM (4 C_H_ exons), IGHD (8 C_H_ exons), IGHX (4 C_H_ exons) and IGHY (4 C_H_ exons). All C genes encode for a transmembrane (TM) and intracellular exon (IC). The 10 exons encoding for IGHD span over 0.228 Mbp (**Figures 1C, D**).

In *X. tropicalis*, an additional C gene, IGHF, is downstream of the IGHY gene. Is composed of two C_H_ exons separated by a hinge exon, followed by the transmembrane and intracellular region exons (8). We identified a syntenic region coding for a partial reading frame homologous to a single C-type Ig domain (PF07654.14), but no evidence of additional C_H_ exons or transcriptional activity within this interval, indicating that in *A. mexicanum*, IGHF is a pseudogene (**Figures 1C–E**).

Class switch recombination is initiated by cytidine deamination mediated by the Activation Induced Cytidine Deaminase (*AICDA*), which preferentially uses the 5’-AGCT-3’ motif as a deamination hotspot (23). Based on the density of occurrence of such motif we identified two highly enriched regions (z-score > 4) (**Figure 1C**; **Supplementary File 1**; **Figures S2A, B**). As expected, the first corresponds to the Sμ region and is located within the 36.93 - 36.942 Mbp interval between the IGHJ cluster and the first IGHM exon. The second region corresponds to the Sχ region and was located within the 37.517 - 37.532 Mbp interval, upstream of the first IGHX exon (**Figure 1C**; **Supplementary File 1**; **Figure S2C**).

No 5’-AGCT-3’ motif enrichment was found upstream of the IGHY or IGHF pseudogene (**Figure 1C**; **Supplementary File 1**; **Figure S2D**). Interestingly, a conspicuous enrichment of the 5’-RGYW-3’ in the direct strand and the 5’-WGCW-3’ palindrome was located in the 37.837 - 37.852 Mbp interval upstream of the IGHY first exon, which corresponds to the Sυ region (**Supplementary File 1**; **Figure S2D**). Further characterization of RGYW motifs revealed that the ACTG motif is also abundant in Sμ but depleted in Sυ. The AGTA motif was abundant in Sυ (predominantly in the reverse strand) and depleted in Sμ and Sχ (**Supplementary File 1**; **Figures S3A, B**). Together, these results indicate that the composition of AID targets may vary within S regions. Except for the absence of the Sφ region, the occurrence of switch regions follows a similar pattern as in *X. tropicalis* (8).

### 2.3 IGHJ, IGHD and IGHV genes

The IGHJ cluster is composed of nine functional IGHJ segments and one pseudogene. Functional IGHJ segments encode for the canonical WGXG motif (di-glycine bulge) and have a 23-bp spacer and highly conserved heptamer and nonamer in their J-RSS. The pseudogene (IGHJ10) is the most proximal to IGHM and lacks a conserved heptamer and nonamer in the RSS (**Supplementary File 1**; **Figure S4**).

Within the 36.6 - 36.8 Mbp interval, upstream of the IGHJ *locus*, we found the IGHD *locus* composed of only four IGHD genes, flanked in both directions by 12 bp spaced RSS’s. The length of three IGHD genes was 11 bps, and one was 13 bp. All IGHD genes were G+C rich, lacked stop codons in the 6 reading frames, and showed partial identity to previously described IGHD core sequences (24) (**Supplementary File 1**;s **Figure S5**).

We found 99 IGHV genes, of which 88 map in chr13q, and the remaining are located in unmapped scaffolds (**Table 1**). Based on the analysis of the functionality of the coding sequence, the presence of RSS’s and in-frame exon-exon junctions defined by RNA-seq, we classified 47 IGHV genes as functional, 21 as ORF’s and 31 as pseudogenes (**Supplementary File 2**, **Table S2**). Three IGHV segments (IGHV_083, IGHV_084, and IGHV_085) are pseudogenes located outside the IGH *locus*, specifically within the class II MHC *locus* in the telomeric region of chr13q (464.8 - 465 Mbp) (**Supplementary File 2**; **Table S1**) (19). As expected, most IGHV segments had a recognizable 23 - bp spacer in their 3’ RSS. However, three apparently functional IGHV segments mapped to chr13q (IGHV_034, 035, and 055) had a 12 - bp spacer between their apparently functional RSS, so we labeled them as ORF’s for violating the 12/23 rule (**Supplementary File 2**; **Table S1**).

**Table 1:** Summary of IGHV and IGLV genes.

		Functional	(%)^#^	ORF	(%)^#^	Pseudogene	(%)^#^	Total
**IGH *locus***	**Chr13q**	43	(43)	20	(20)	25	(25)	88
	**non-mapped**	4	(4)	1	(1)	6	(6)	11
	**Total**	47	(47)	21	(21)	31	(31)	99
**λ** ***locus***	**Chr10p**	44	(62)	5	(7)	21	(30)	70
	**non-mapped**	1	(1)	0	(0)	0	(0)	1
	**Total**	45	(63)	5	(7)	21	(30)	71
**σ** ***locus***	**Chr 1p**	5	(83)	1	(17)	0	(0)	6
	**non-mapped**	0	(0)	0	(0)	0	(0)	0
	**Total**	5	(83)	1	(17)	0	(0)	6
**κ** ***locus***	Not found. Syntenic non-Ig genes found in chr6q: 1.4-1.5 Gb							

^#^Percentages are based on the total number of genes per *locus*.

IGHV segments in tetrapods are classified in three major phylogenetic clans based on sequence conservation of the framework regions (2). Phylogenetic analysis of axolotl functional IGHV using human and mouse IGHV sequences as reference revealed 24 segments belonging to clan II, 19 segments belonging to clan III, and the absence of clan I segments (**Supplementary File 1**; **Figure S6**). Four IGHV genes (IGHV_070, 071, 077 and 082) could not be assigned to a particular clan (**Supplementary File 2**; **Table S1**). IGHV ORF’s also were assigned to clan II (n = 11) and clan III (n = 4), but none to clan I, and seven could not be assigned to any clan. Of the eleven IGHV families described by (25) in *A. mexicanum*, we found functional representatives for all but family VH6 and VH9 (**Supplementary File 2**; **Table S1**). Four genes, IGHV_005, IGHV_039, IGHV_063, and IGHV_065, which belong to the VH8 family, contain a triple Cys in the CDRH1, which appears to be a unique feature of urodeles (15). Moreover, seven IGHV genes (IGHV_051, 056, 057, 058, 061, 062, and 082) could not be assigned to any family (**Supplementary File 1**; **Figure S7**).

### 2.4 *A. mexicanum* light chain *loci*

**Figure 2:**
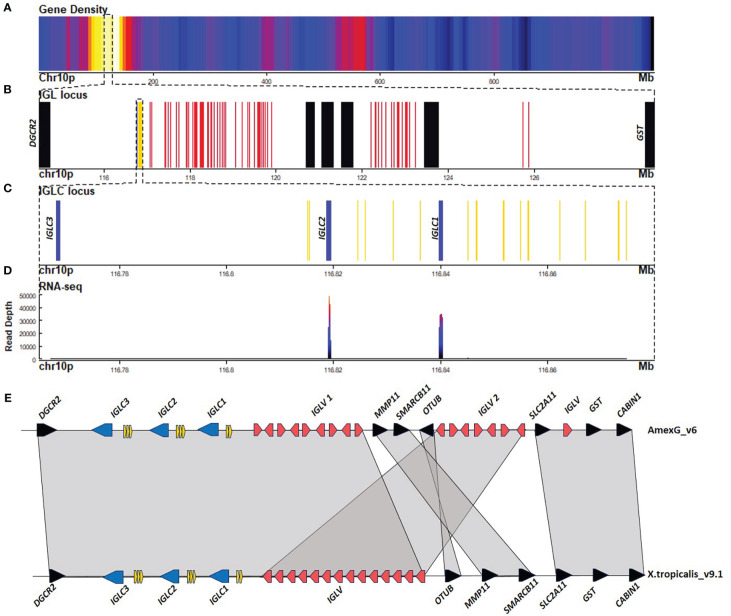
The lambda light chain *locus* is located in the centromeric portion of chr10p (116.7 - 125.8 Mbp). **(A)** Gene density plot of chr10p where the IGL *locus* is encoded (box). Dark blue colors indicate low gene density. **(B)** Zoom of the whole IGL *locus* (114.5-128.8 Mbp). Non-Ig genes (black) in proximal flank *DGCR2* gene, and distal flank *GST*. Zoomed view of IGLC genes (blue), IGLV genes (red), and IGLJ (yellow). **(C)** Zoomed view (116.76-116.88 Mbp) of the IGHC gene cluster. **(D)** Spleen RNA-seq coverage histogram. Note that there is no expression in Cλ3. **(E)** Schematic representation of immunoglobulin lambda *locus* in *A. mexicanum* genome (chr10p:116-124 Mbp) and *X. tropicalis* (chr1:144.7-144.9 Mbp). Color code as in A *X. tropicalis* displays the canonical architecture, in which the V, J, and C clusters are in the same orientation. Note that in *A. mexicanum*, IGLV genes are in at least two different clusters (IGLV1 and IGLV2), separated by the *MMP11*, *SMARCB11*, and *OTUB* genes, which in *Xenopus* are located in the distal flank. Also, note that the *OTUB* gene in axolotl is inverted. Moreover, as in the IGHV *locus*, IGLV segment orientation in axolotl is intercalated, which is atypical. VJ recombination involving the more distal IGLV segments would delete the *OTUB*, *SMARCB11*, *MMP11*, and the *SLC2A11* gene cluster. Not on a scale.

**Figure 3:**
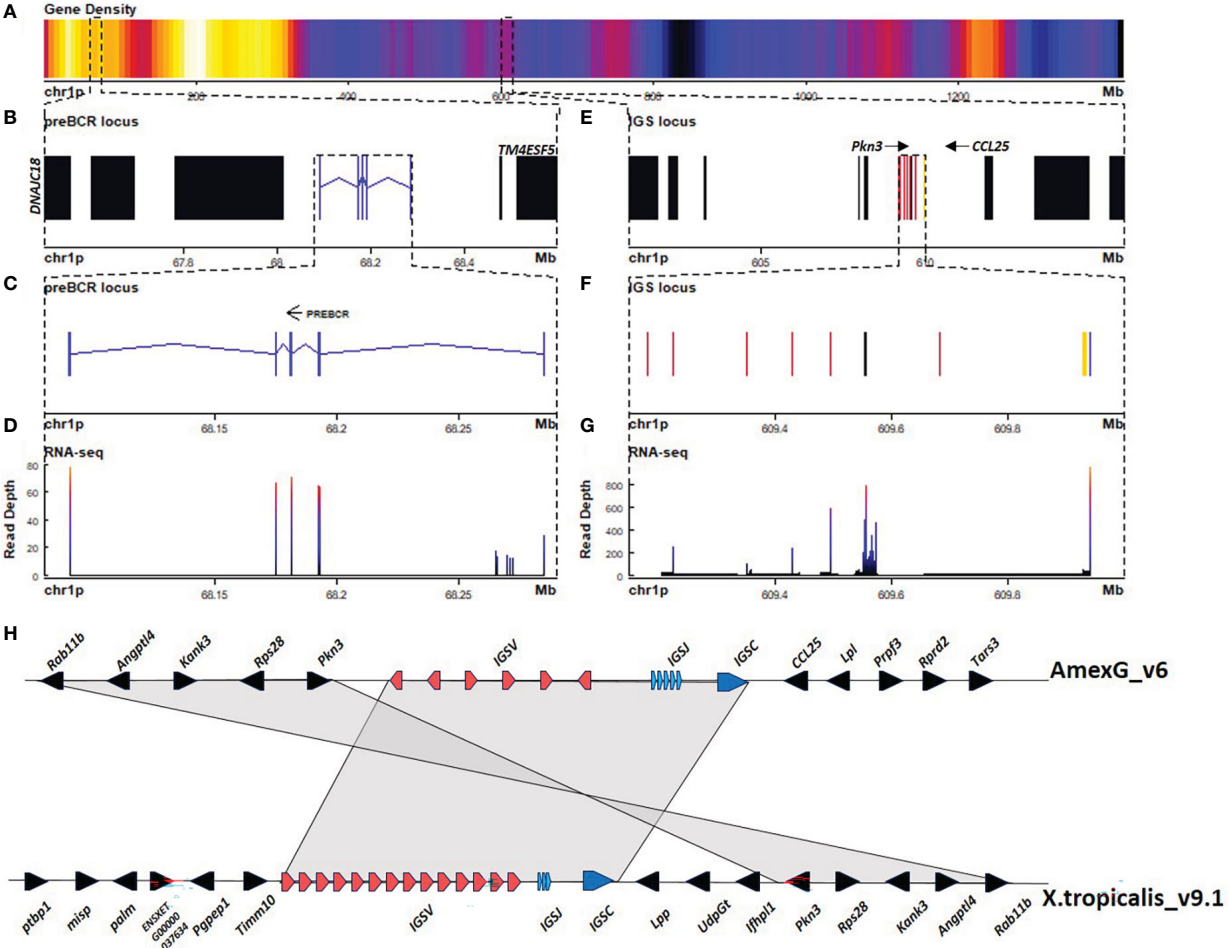
The sigma light chain *locus* is located in the centromeric portion of chr1p (609.1 - 609.2 Mbp). **(A)** Gene density plot of chr1p where the IGS and the putative surrogate light chain (SLC) *loci* are encoded (box). Dark blue colors indicate low gene density. **(B)** Zoom of the whole putative SLC *locus* (67.5 - 68.6 Mbp). Non-Ig genes (black) in proximal flank *DNAJC18* and distal flank *TM4ESF5*. Zoomed view of IGLC genes (blue). **(C)** Zoomed view (68.08 - 68.29 Mbp) of the putative SLC gene. **(D)** Spleen RNA-seq coverage histogram. **(E)** Zoom of the whole IGS *locus* (601 - 616 Mbp). Non-Ig genes (black) in proximal flank *RAB11B* gene and distal flank *PRPF3*. **(F)** Zoomed view of IGSC genes (blue), IGSV genes (red) and IGSJ (yellow) (609.15 - 610 Mbp) **(G)** Spleen RNA-seq coverage histogram**. (H)** Schematic representation of Immunoglobulin sigma *locus* in *A. mexicanum* genome (v6) (chr1p:609 - 610 Mbp), and *X. tropicalis* (chr1: 82.1 - 82.22 Mbp). Color code as in A. *X. tropicalis* displays the canonical architecture, in which the V, J and C clusters are in the same orientation. Note that in *A. mexicanum*, IGSV gene orientation in axolotl is intercalated, which is atypical. Non-Ig genes in the downstream flank are different in *A. mexicanum* and at least the *PRPF3*, *RPRD2* and *TARS3* genes are located in chr8 106.9 – 107.1 Mbp in *X. tropicalis*. Also, non Ig genes in the upstream flank correspond to the downstream flank in *X. tropicalis*. It is possible that this chromosomal configuration is correct, however it is also possible that the IGS *locus* may be inverted so that the downstream flank is syntenic with *X. tropicalis*. Not on scale.

**Figure 4:**
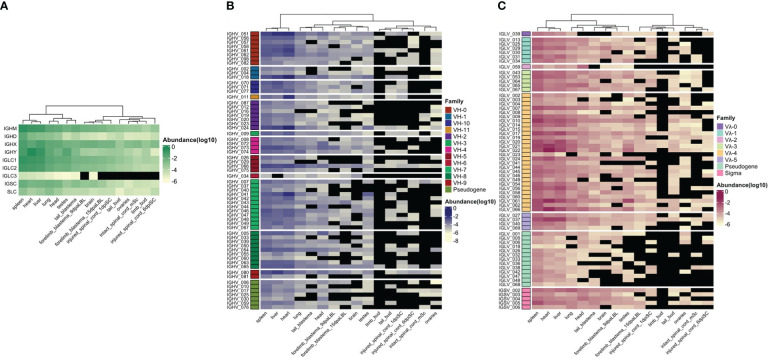
Relative immunoglobulin gene transcription (Log10 relative abundance). Publicly available RNA-seq data (SRA: SRP101842) (17) was used to estimate relative gene transcription in 13 adult tissues (spleen, liver, heart, lung, testes, ovary, brain, head, 9 and 15 day post - amputation forelimb blastemas, tail blastema, intact, 1 and 6 days post-injured spinal cord), limb and tail buds. A hierarchical clustering by column (tissue) was performed. **(A)** Immunoglobulin C region relative transcription. **(B)** IGHV relative transcription. Each IGHV gene is grouped by IGHV family (rows). **(C)** IGLV and IGSV relative transcription. Each IGLV segment is grouped according family.

We found three Ig light chain *loci*: A large 9 Mbp *locus* in chr10p:116.7 - 125.8 Mbp (**Figure 2**), a 0.1 Mbp *locus* at chr1p:609.1 - 609.2 Mbp (**Figures 3A, E–H**), and a third 0.2 Mbp *locus* at chr1p: 68.0 - 68.2 Mbp (**Figures 3A–D**). The larger *locus* in chr10p corresponds to the lambda (IGL) and is similarly structured as in *X. tropicalis*, also referred to as the type III light chain *locus* (26, 27). It is composed of a tandem of three Cλ genes and their corresponding IGLJ gene clusters, followed by a single IGLV cluster (**Figures 2A, B**). The three Cλ genes encode for the characteristic Lys23 and Lys60 unique to Cλ, which are consistent with Cλ cladistic markers (28, 29) and cluster together with human and *X. tropicalis* Cλ protein sequences (**Figure 2C**; **Supplementary File 1**; **Figure S8**). Only Cλ1 and Cλ2 were transcribed in spleen RNA (**Figure 4A**).

IGLJ cluster 1 (upstream of Cλ1) is the largest cluster with 9 IGLJ gene segments (IGLJ1.1-1.9), all functional but IGLJ1.6, which lacks a conserved heptamer at the RSS. The second IGLJ cluster comprises four functional IGLJ segments (IGLJ2.1-4). The third cluster comprises only two functional IGLJ segments (IGLJ3.1-2). Eleven out of 15 IGLJ segments encode for the canonical motif FGXG characteristic of IGLJ and IGKJ. In the remaining four, the Phe is replaced by Ile (IGLJ2.1 and 3.1) or Leu (IGLJ1.1 and 2.4) (**Supplementary File 1**; **Figure S9**). In contrast with IGKJ or IGSJ gene segments, all IGLJ are flanked by 12 bp-spaced RSS’s (**Supplementary File 1**; **Figures S9**, **S10**).

The cladistic marker analysis (28) confirmed the identity of the IGLV segments, which includes the sequence gap between position 7 (FWR1), presence of gaps at positions 41a, 46a, and 46b (FWR3), presence of Ala53 instead of S/T (FRW3) and the DEAD motif in positions 64-67. Additionally, IGLV gene segments were flanked by 23 bp - spacer RSS (**Supplementary File 1**; **Figure S11**). The IGLV cluster comprises 71 IGLV genes, of which 45 appear functional (**Table 1**; **Supplementary File 2**; **Table S3**). Phylogenetic analysis revealed that 41 functional segments belong to the four previously described IGLV families (29), but IGLV_040, IGLV_069, and IGLV_037 are likely to belong to a novel family (**Supplementary File 1**; **Figure S12**).

As in the IGH *locus*, many IGLV segments are intercalated in opposing directions. In *X. tropicalis*, the IGL *locus* is flanked by the *DGCR2* gene on the centromeric flank and the *OTUB* genes in the telomeric flank. While in axolotl *DGCR2* also flanks IGL on the centromeric end, *OTUB, MMP11*, and *SMARCB11* split the IGLV cluster in two, but overall synteny (*SLC2A11, GST*, and the *CABIN1* genes) is maintained in the telomeric flank (**Figures 2B, E**).

The second light chain *locus* is composed of only 6 V genes (5 functional), flanked by a 12 bp-spaced RSS, 5 J genes flanked by a 23 bp-spaced RSS, and a single C gene composed of 2 exons (**Figures 3E–F**; **Table 1**; **Supplementary File 2**; **Table S1**; **Table S4**). Is similarly structured to the *X. tropicalis* IGS *locus* (27), including synteny in one flank (*ANGPTL4*, *RAB11B*, and *MARCHF2* genes) (**Figure 3H**). Cladistic analysis is consistent with the identity of this *locus* as the σ *locus*, such as the presence of Thr7 (FWR1), absence of gap in positions 41a and 46b (FWR3), the presence of Tyr53, and absence of DEAD motif (64–67) (FWR3) in V genes, as well as the presence of FSXS motif instead of the FGXG (di-Glycine bulge) motif of IGKJ or IGLJ (**Supplementary File 1**; **Figure S10, S11**).

The third light chain *locus* is in chr1p (68.1 – 68.3 Mbp) contains a single gene composed of five exons encoding for a 307-residue polypeptide. The first exon encodes for a signal peptide, exon 2 encodes for an immunoglobulin V-set domain (PF07686), exon 3 encodes for an immunoglobulin Cλ-set domain (PF07654), and exons 4 and 5 encode for the C-terminal region and the 3’ UTR, with no homology identified by BLASTP to the nr NCBI database.

Structural analysis of the V-type domain, revealed the conservation of Gly16 (FWR1) and Phe76, Thr 90, Ile 91, and Glu99 (FWR3) (IMGT numbering), which are common in Ig light chains, but not in heavy chains. More importantly, we found in β strand G, sequence homology to Jλ di-glycine bulge (FGDGTQVIYR), common in acquired immunity antigen receptors. In the C-type Ig domain, we found a Cys residue at position 250 conserved in light chain C regions, involved in H+L chains disulfide pairing. Similarly, Pro33 and His90, which are common to κ and λ light chain C-type Ig domains are conserved in positions 178 and 234, respectively. Moreover, characteristic of Cλ, Lys23 is substituted by Arg (pos 166), but Asp31 (pos 175) and Lys60 (pos. 203) are conserved (**Supplementary File 1**; **Figure S8A**). Based on the predicted protein and gene structure, we suggest that this gene encodes for the surrogate light chain (SLC) of the preB cell receptor.

In *X. tropicalis*, a substantial portion of the light chain repertoire is composed of κ light chains, also referred to as ρ (rho) *locus* (27). It is encoded in the telomeric tip of chr1 (2-2.8 Mbp). We did not find evidence of the κ *locus* in *A. mexicanum*, although non-Ig the orthologs in the centromeric flank (*SUCLG1)* and telomeric flank (*ADRA1D* and *RNF24*) of the *X. tropicalis* κ *locus* were found in *A. mexicanum* chr6: 1444 – 1503 Mbp (**Supplementary File 1**; **Figure S13**).

### 2.5 Transcriptional analysis of Ig *loci*

To gain insight into tissue immunoglobulin gene expression in the axolotl, we mapped publicly available RNA-seq data to Ig *loci* to obtain relative expression based on reading counts per gene (**Figure 4**). Between 0.001 to 1.1% of the transcriptome mapped to Ig *loci*. Surprisingly, of all mapped reads to the Ig *loci*, 37% were derived from the heart, followed by 29% and 22% from the spleen and liver, respectively. The lung contributed to 4%, followed by 3% and 2% from the head and testes. Each of the remaining libraries (brain, intact and injured spinal cord, ovary, tail, and forelimb blastemas) contributed with 1% or less (**Supplementary File 2**; **Table S5**).

IGHM gene expression was dominant in spleen, liver and heart, with considerably lower expression levels in nervous system and regenerating tissues. IGHD gene was mainly expressed in spleen. Although IGHX and IGHY expression was also predominant in spleen, liver and heart, IGHX peaked in spleen and liver, whereas IGHY was predominantly transcribed in heart and in a lesser degree in testes (**Figure 4A**).

Similar to IGHM and IGHX, IGLC1 and IGLC2 were highly transcribed in spleen, liver and heart, with less transcription in the remaining tissues. Transcription of IGLC3 was null or negligible in all tissues. Transcription of IGSC was comparatively lower than IGLC and predominated in spleen, liver, heart and lung. Similarly, transcription of the putative SLC gene was comparatively lower than for heavy and light chain genes, and was predominant in spleen, lung and liver (**Figure 4A**).

As expected, IGHV, IGLV, and IGSV gene transcription mirrored IGC transcription with predominant transcription in spleen, liver, and heart (**Figures 4B, C**). We found some degree of transcription of IGHV genes of all families. IGHV family transcription was more evenly distributed in the spleen, although IGHV_051 (unclassified family) and IGHV genes of the VH7 and VH8 family predominated. Transcription of IGHV_051, followed by VH7 and VH8 family members also predominate in the liver. Contrastingly, IGHV_18 (VH1 family) and IGHV_24 (VH2 family) transcription markedly dominated in the heart, followed by IGHV_51. The only member of the VH11 family, IGHV_011, was also highly transcribed in the heart.

As for IGLV gene transcription, we found that all families were transcribed, although Vλ4 showed dominant transcription, mainly because it has more genes. As for IGHV genes, spleen Vλ family transcription was more evenly distributed, in contrast to heart and liver transcription, in which IGLV_021, IGLV_061, IGLV_066 (family Vλ4) and IGLV_037 (Vλ5) predominated (**Figure 4C**). Spleen, liver and heart transcription of IGLV_059, the only member of Vλ2 family, was low compared with its expression in the head. IGSV transcription was mainly dominated by IGSV_005, and IGSV_002 and represented a minor fraction of the whole light chain transcriptome (**Supplementary File 2**; **Table S5**).

### 2.6 Implications of genome size in the architecture of IGH *locus* in *A. mexicanum*

**Figure 5:**
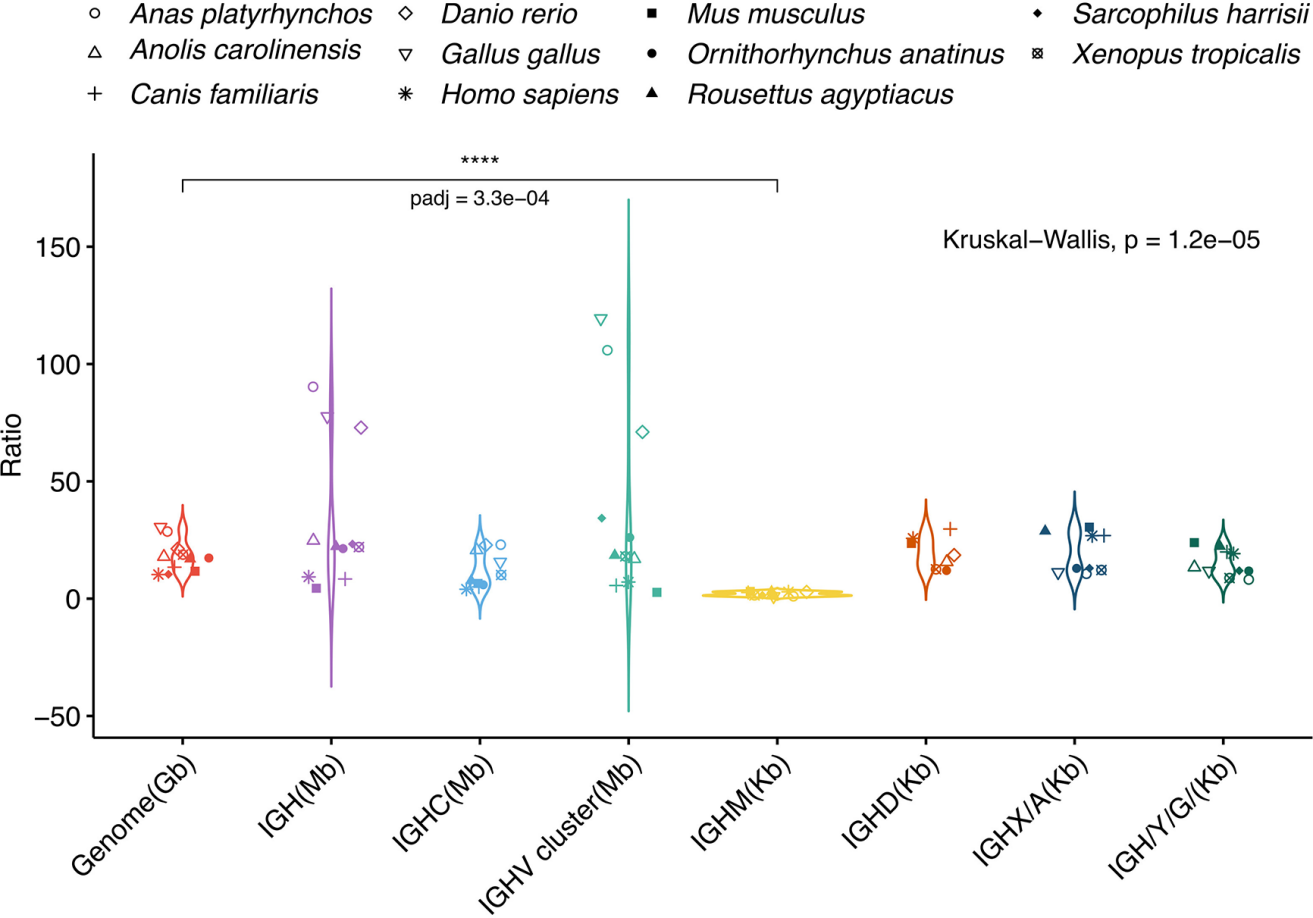
IGHM gene length in *Ambystoma mexicanum* is evolutionarily constrained. *A. mexicanum* genome (orange), IGH (purple), IGHC (light blue), IGHV cluster (light green) and individual IGHC gene length size ratios are plotted (from left to right). Comparisons include four placental mammals (human, mouse, dog and a bat), two non-placental mammals (Tasmanian devil and platypus), two bird species (chicken and duck), and one reptile, amphibian and teleost fish. Only the IGHM *A. mexicanum*: species size ratio was smaller to the respective genome size ratio (Kruskal-Walllis test, Dunnet multiple comparison correction (p = 0.0003).

The *A. mexicanum* genome is 10-30-fold larger than most vertebrate genomes. We asked if the axolotl IGH *locus*, the IGHC cluster, and individual IGHC gene lengths were proportional to the genome size difference in other vertebrates, including placental and non-placental mammals, birds, a reptile and a teleost fish (*D. rerio*) (**Figure 5**). The axolotl IGH *locus*, the IGHC and IGHV clusters and individual IGHC genes are roughly proportional to the corresponding genome sizes (**Figure S14**). The large variation observed at the IGHC and IGHV clusters is attributed to a highly compact IGH *locus* in zebrafish and birds (chicken and duck) (**Supplementary File 1**; **Figure S14**; **Supplementary File 2**; **Table S6**). Interestingly, while IGHD, IGHX (or IGHA), and IGHY (IGHG/IGHE) were 8 - 12 times in most species, IGHM size fold difference was significantly lower than expected form genome size difference (p-value = 0.0003; **Figure 5**). Axolotl IGHM is 12.9 Kb long whereas IGHD, IGHX and IGHY are 228.5, 123.1 and 109.3 Kb long, respectively. Axolotl IGHM length is only 1.4 to 3-fold larger than the corresponding ortholog in all species tested, with the exception of birds (**Supplementary File 1**; **Figure S14**; **Supplementary File 2**; **Table S6**). These results suggest that in the axolotl, some regions of the IGH *locus*, and particularly the IGHM, but not IGHD gene intron length, is evolutionarily constrained.

### 2.7 V-intron length

**Figure 6:**
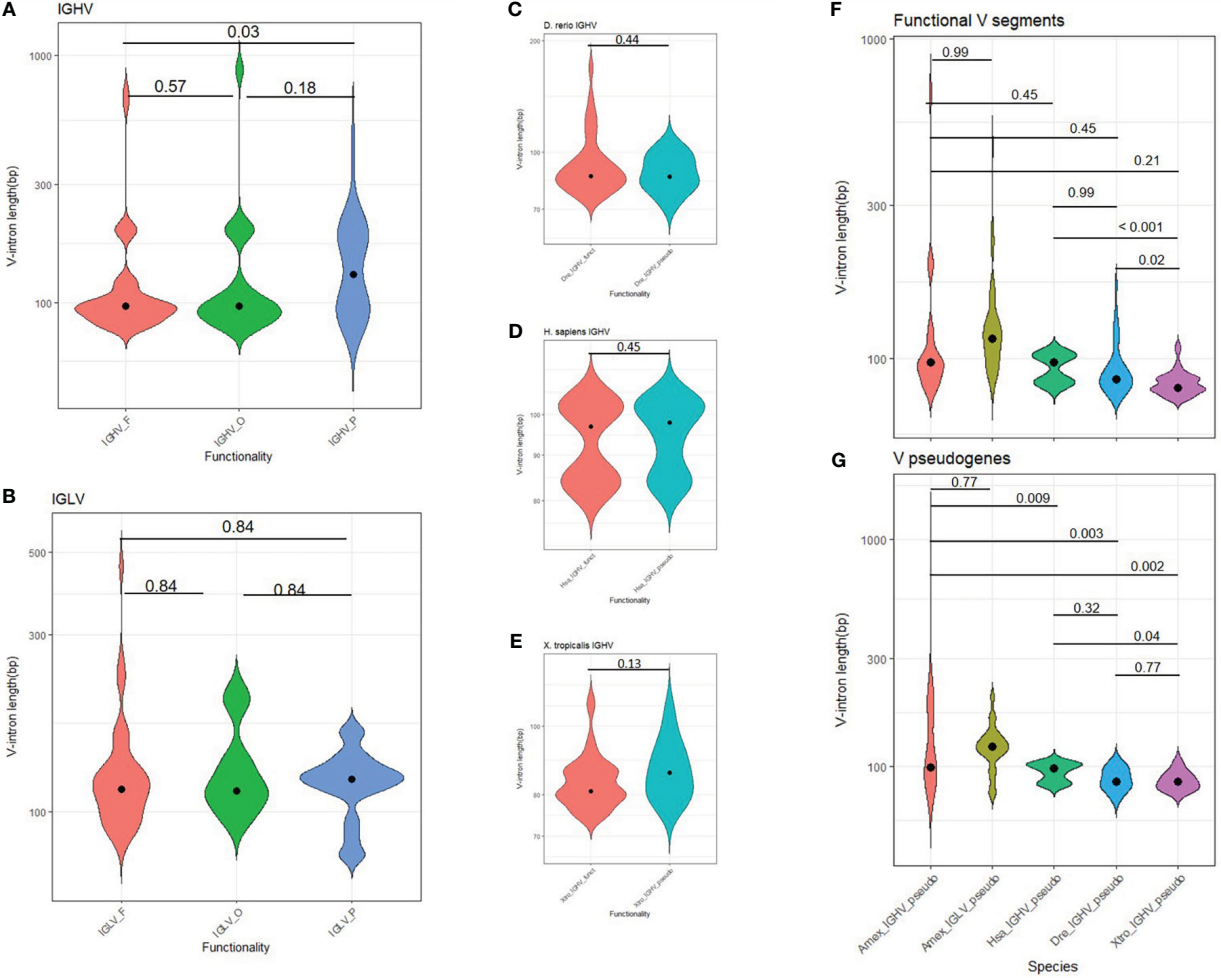
Distribution of V intron length in tetrapods. **(A)** The heavy chain and **(B)** lambda light chain *locus* in *A. mexicanum* according to their functionality class (functional, ORF or pseudogene). In *A. mexicanum*, intron length in functional IGHV genes was shorter than in IGHV pseudogenes. No differences were observed in the lambda *locus* and **(C)** *D. rerio*, **(D)** *H. sapiens*, and **(E)** *X. tropicalis*. V introns in functional IGHV *A. mexicanum* were not larger than functional *A. mexicanum* IGLV genes, or **(F)** functional IGHV of other tetrapods, however, V introns in **(G)** non-functional *A. mexicanum* IGHV genes were larger than other tetrapods. Statistical analysis for multiple comparisons **(A, B, F, G)** was performed with the robust ANOVA One-Way Trimmed Means Comparisons, with a trimming level of 5% (tr = 0.05), followed by the *post hoc* Lincon test (30). For two sample comparisons **(C-F)**, Yuen’s robust Tests for Two Independent Groups were performed with a trimming level of 5%. The violin area is scaled for comparability. The median is shown as a black dot.

The first exon of each V gene encodes for a signal peptide that allows the BCR or TCR to follow the secretory pathway. In axolotl, regardless of V gene functionality, V-intron median length for IGHV and IGLV was 97 and 115 bp, respectively (**Table S6**). However, a subset of 24 IGHV and 11 IGLV genes had abnormally large V-introns (> 150 bp) (**Figures 6A, B**; **Supplementary File 2**; **Table S1**, column L). We reasoned that genome enlargement by genome drift would be accompanied by increasing V-intron lengths unless intron size is functionally constrained. By measuring the distribution of V-intron length, we observed that the V introns of functional *A. mexicanum* IGHV genes were shorter than in IGHV pseudogenes (P = 0.037; **Figure 6A**). No differences in V intron length were noted between functional and non-functional IGLV genes (P = 0.84; **Figure 6B**) or IGHV introns in *D. rerio* (P = 0.45; **Figure 6C**), *H. sapiens* (P = 0.44; **Figure 6D**), and *X. tropicalis* (P = 0.13; **Figure 6E**). However, comparison of *A. mexicanum* V-intron length IGHV across species revealed no differences in functional IGHV genes (P > 0.05; **Figure 6F**), whereas non-functional IGHV of *A. mexicanum* were larger than human (P = 0.009), zebrafish (P = 0.003) and frog (P = 0.002) (**Figure 6G**, **Supplementary File 2**; **Table S7**).

To further explore a relationship between V-intron length and functionality in *A. mexicanum*, we tested if the observed number of long introns (> 150 bp) were more frequent in non-functional IGHV and IGLV segments than expected by chance. A Fisher’s exact test revealed that the odds of an IGHV gene with a long V-intron being non-functional are 3.3 higher than its short V-intron counterpart (P = 0.018, CI95: 1.1, 10.7) (**Supplementary File 1**; **Figure S15A**). In contrast, for the IGLV *locus*, there were no differences in the observed and expected frequencies of short and long V-introns according to functionality (P = 0.73; OR = 0.63, CI95: 0.09, 3.0). Overall, these results indicate that in *A. mexicanum*, an increase in V-intron length is evolutionarily constrained in IGHV but not IGLV (**Supplementary File 1**; **Figure S15B**).

### 2.8 IGHV and IGLV intergenic lengths are shorter

We reasoned that an increase in *A. mexicanum* genome size would not impact the Ig *loci* architecture unless there are functional constraints for Ig *loci* enlargement. The size difference of the IGHV gene cluster in *A. mexicanum* (17, 7, and 70 times larger than *X. tropicalis*, human, and zebrafish, respectively) is roughly proportional to its genome size (**Figure 5**; **Supplementary File 2**; **Table S6**), but this could be highly influenced by IGHV gene number.

**Figure 7:**
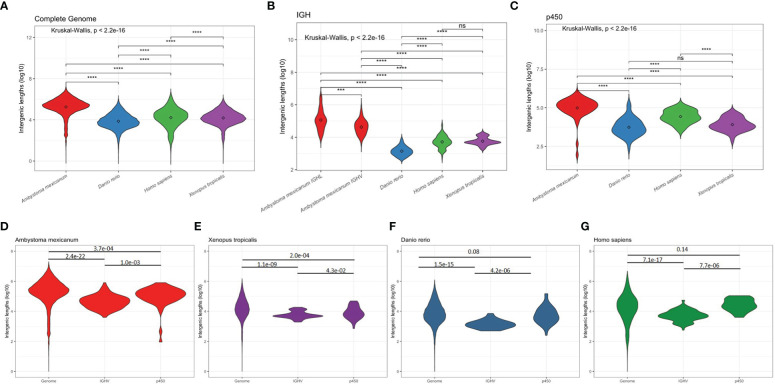
Intergenic distances by species. Violin plots depicting overall coding to coding gene intergenic distance distribution across the whole genome **(A)**, between IGHV segments **(B)**, and between cytochrome p450 family gene clusters **(C)** in **(D)** *A. mexicanum* (red), **(E)** *X. tropicalis* (purple), **(F)** *D. rerio* (blue), and **(G)** *H. sapiens* (green). Kruskal-Wallis rank sum test followed by Dunn’s multiple comparison tests with Benjamini – Hochberg p adjustment.

We further compared functional IGHV and IGLV intergenic length distribution in *A. mexicanum*, *H. sapiens*, *D. rerio*, and *X. tropicalis*. As a reference for comparison, we used the whole genome coding genes and cytochrome *p450* family (a non-Ig gene family commonly encoded in gene clusters) intergenic distance. As expected, *A. mexicanum* whole-genome (**Figure 7A**), IGHV and IGLV (**Figure 7B**), and *p450* intergenic lengths (**Figure 7C**) were larger than their corresponding distributions in all tested species. In *A. mexicanum*, IGLV intergenic lengths were larger than IGHV (**Figure 7B**). Whole-genome intergenic lengths were larger than IGHV (P = 2.4e-22) and *p450* (P = 3.7e-04), and IGHV were smaller than *p450* (P = 1.0e-03) (**Figure 7D**). Although *p450* intergenic lengths in *H. sapiens* and *D. rerio* were no different than whole-genome (**Figures 7F, G**), *A. mexicanum* and *X. tropicalis* were smaller (**Figures 7D, E**). Nevertheless, we observed that in all species, IGHV intergenic lengths were consistently smaller than the whole-genome and *p450* (**Figures 7D–G**), indicating that the IGHV cluster size is constrained by natural selection.

## 3 Discussion

The availability of a high-quality genome draft of *A. mexicanum* assembled at the chromosome level enabled us to evaluate Ig *loci* organization and its relation to previous functional and structural analysis of the axolotl humoral immune response (24, 25, 31). Genomic analysis provided the opportunity to investigate if the Ig *loci* grow in parallel with the rest of the genome or if there are functional constraints that limit Ig *loci* enlargement.

From a comparative immunogenomic perspective, we found loss of function of the IGHF gene, lack of IGHV segments belonging to the tetrapod clan I, and loss of the IGK *locus*. Previous studies revealed limited VH junctional diversity (24, 32), which we confirmed to be the result of only four IGHD segments of limited length (11 and 13 bp long). From a genome evolution perspective, we found an unusual increase of V-intron size in a subset of IGHV and IGLV segments and that although Ig *loci* size has increased, the IGHM gene and the IGHV and IGLV intergenic distances have not grown at the same rate as the rest of the genome.

We identified an atypical configuration of the IGH *locus*, particularly regarding the position of the IGHJ and IGHC genes upstream of the IGHV cluster (**Figure 1**). This unusual architecture would be incompatible with B cell maturation and antibody production because V(D)J recombination involving IGHV segments downstream IGHJ *locus* would delete the IGHC cluster. In the case of the IGL *locus*, VJ recombination involving the more distal IGLV segments would delete the *OTUB*, *SMARCB11*, *MMP11*, and the *SLC2A11* gene cluster. Moreover, V gene clusters in alternating coding directions would be incompatible with chromatin extrusion during V(D)J recombination due to frequent convergence of CBE’s (33) (see below). We propose that such inconsistencies may be the result of local scaffold orientation errors attributable to the inherent complexity and genome size, combined with the repetitive nature of the Ig/TCR *loci*, despite the enormous technological effort involving long-read sequencing, SNP segregant and fiscal mapping, and Hi-C mapping (17–19).

Apart from such inconsistencies, the overall organization of the Ig *loci* in *A. mexicanum* is similar to the anuran *X. tropicalis*, and we confirmed studies describing the main antibody classes, IgM and IgY (34), IgX (35) and IgD (36). Interestingly we demonstrate that IGHF is a pseudogene. IGHF in *Xenopus* is one of the earliest examples of an antibody hinge region encoded by an exon (8). The functional implications of IGHF loss of function axolotl are unknown but worthy of further research. Transcriptome public data revealed that most heavy and light chain transcription occurred in spleen, liver and unexpectedly in heart, where predominant H chain class transcription was IGHY. The high transcription of IGHY in the heart could be the result of IgY –secreting plasma cells homing in the heart, or the presence of more complex cardiac lymphoid tissue aggregates. A recent scRNA-seq study showed a significant presence of immune cells, including B cells in cardiac tissue of neotenic axolotls, which increases on metamorphosed axolotls (37).

Studies by Charlemange’s group anticipated that most if not all light chains in axolotl were λ chains (29). Extensive homology-based sequence searches in the previous genome version (V3) and the current version (V6) revealed the absence of the IGK or ρ *locus* in axolotl. Search for cladistic markers, RSS configuration, and overall *locus* architecture allowed us to unambiguously define that the only light chain *loci* identified in current assemblies correspond to λ, σ, and the putative surrogate light chain (SLC), a component of the preB cell receptor. Genome search for IGK in other caudate amphibians may reveal if IGK loss is restricted to axolotl.

The surrogate light chain is a non-recombined light chain that associates with successfully recombined μ chains and is expressed only during proB to preB cell development at relatively low levels. We found a gene in chr1p (68.1 – 68.3 Mbp) encoding for a 307 residue IgSF protein with structural features of light chains, including a conserved Cys 104 for heavy chain pairing and the di-glycine bulge at the G β strand of the V domain, whose transcription correlates anatomically with axolotl hematopoiesis in spleen and liver (38). Based on these structural and functional findings we proposed that this is the axolotl surrogate light chain gene, but more experimentation is required to provide a definite proof. Noteworthy, in contrast to human and mouse where the SLC is encoded by two genes, VPREB and λ5 (39), the putative SLC is axolotl is encoded by a single gene.

Larger introns may be biologically costly by increasing the investment of nucleotides and time, compromising the fidelity of the resulting mRNA, and increasing the space for allelic variation and aberrant splicing (40). Recent *in situ* studies in human cells show that splicing efficiency is not affected by exon number and intron lengths (41, 42). Comparative analysis of intron length distribution in axolotl revealed that introns of genes involved in developmental processes are shorter than non-developmental genes, suggesting an evolutionary constraint that favors higher transcriptional rates (17). Similarly, we observed that the axolotl IGHM size, but not other IGHC genes size is very stable despite the wide variation in genome size. As the size of the IGHM coding exons is constant, our results indicate an intron size constraint favoring IGHM transcription levels or regulation.

Intron size also varies within each gene, and constitutive exons are flanked by shorter introns than alternatively spliced exons (43). The first exon of each V gene encodes for the majority of the signal peptide (L-PART1) in immature heavy or light chain polypeptides and, as such, are constitutive exons. Except for a subset of abnormally large V-introns found in the axolotl (up to 800 bp), we found that V-intron length is usually short (80-100 bp), as in the remaining species tested. Also, it is more likely that IGHV pseudogenes have abnormally large V-introns than their functional counterparts. Moreover, only in the axolotl, V-intron length is larger in IGHV pseudogenes than in functional IGHV genes. However, this association was not found in the lambda *locus*. The axolotl putative SLC gene spans over 195 Kb, and the V-intron is 93 Kb long. Assuming a similar RNApolII elongation rate to humans (41), a SLC precursor RNA would take 51 min to synthesize. We propose that V-intron lengths at the IGH *locus*, but not the light chain and SLC *loci*, are constrained by evolution to favor stringent transcriptional control during preB cell development.

The hallmark of adaptive immunity is the generation of a vast array of clonally distributed antigen receptors generated by V(D)J recombination. Much knowledge has been generated that explains how the RAG recombinase mediates precise double-stranded breaks in V(D)J segments over tens to hundred kilobases apart. Non-coding transcription and chromatin remodeling are crucial (21). More recently, it has been shown that the mouse IGH *locus* is a topologically associated domain (TAD) organized into three chromatin loops or sub-TAD’s required for adequate spatial approximation of IGHV genes to the recombination center by chromatin loop extrusion (33, 44). Chromatin extrusion is mediated by the cohesin ring protein complex by extruding chromatin between convergently oriented CTCF-binding elements (CBE’s). Whole-genome TAD analysis in axolotl revealed that TAD size increase does not affect function negatively (19). We asked whether the size of the IGHV gene cluster is constrained in axolotl. Despite the large size of the IGHV gene cluster (6.7 Mbp), we observed that intergenic IGHV distances are significantly smaller than all genome intergenic distances or *p450* gene clusters, indicating that larger distances impose a functional cost that it does not affect non-coding transcription (required for the initiation), could affect V(D)J recombination *per se* by favoring spurious recombination events due to cryptic RSS´s.

In conclusion, *A. mexicanum* Ig *loci* share the same structure with other tetrapods, however, it shows signs of loss of function such as a large proportion of V pseudogenes and an IGHF pseudogene, loss of the κ *locus*, and a very small σ *locus*. We provide evidence suggesting an increase in genome size may be causally related to this loss of function, particularly in the IGH *locus*, and that Ig *loci* enlargement has been negatively selected, likely to counteract a negative functional effect in transcriptional regulation and/or V(D)J recombination. Systematic functional immunocompetence analysis in amphibians and other vertebrates with large genomes may provide a definitive answer.

## 4 Methods

### 4.1 *A. mexicanum* genome data

The latest version genome sequence of *A. mexicanum* white d/d strain AmexG_v6.0-DD, and the corresponding annotation file AmexT_v47-AmexG_v6.0-DD.gtf.gz was downloaded from https://www.axolotl-omics.org/assemblies (19).

### 4.2 Immunoglobulin *loci* mapping

We used reference IGHV, IGHC, IGLC cDNA sequences obtained IMGT (http://www.imgt.org/) to map the IGH and IGL *loci* using TBLASTX, Exonerate (EST2genome alignment model). Significant hits (e-value < 1.0E^-05^ for BLASTX, score > 100 for Exonerate) were exported as GFF3 files to load them in the Integrative Genomics Viewer (IGV) (45) for visualization and manual curation. Searches were further complemented using HMMER3 option *hmmsearch*, using PFAM immunoglobulin domain *hmm* models (V-set: PF07686.17 and C-set: PF07654.14).

Gene models were refined by mapping transcriptomic data from *A. mexicanum* spleen from public RNA-seq spleen data (project SRP101842, run SRR5341570), downloaded from https://www.ncbi.nlm.nih.gov/sra. For mapping RNA-seq reads from both sources, we used the STAR aligner (46), using only relevant chromosome arms as targets.

### 4.3 Definition of V, D, and J functionality

Functionality was defined based on the IMGT criteria (47). For IGHV segments to be functional (F), the presence of a predicted *in-frame* signal peptide exon/V exon open reading frame (ORF) compatible with a V-domain, the presence of Cys23, Trp41, Trp 52, and Cys104 in the V exon, followed by a *bona fide* RSS (as described above). Spleen RNA-seq data was used to aid in the identification of exon-exon junctions. We consider as non-functional V-ORF (O), the presence of a V-domain ORF in absence of Cys22, Trp36, Trp 47, or Cys104, or the absence of RSS. Frame-shifted V-ORF, interruptions by stop codons, or lack of signal peptide exon was regarded as a V-pseudogene (P).

### 4.4 Recombination signal sequence identification and analysis

Forty-two base pairs downstream of each presumably functional Variable segment were aligned with Clustal X and manually edited according to the vertebrate consensus CACAGTG and ACAAAAACC. A Positional weight matrix was calculated for the 7 and 9-mer, and the spacer length was calculated. A bona fide RSS was considered when the heptamer and nonamer weight score was ≥ half the corresponding maximal score and the spacer length was 23 ± 1 bp long (IGHV, IGHJ, IGLV, and IGSJ) or 12 ± 1 bp long (IGHD, IGLJ, and IGSV).

### 4.5 Switch regions identification

We used the chr13q 35-50 Mbp interval to search in both strands for the occurrence of the AID hotspot motif 5’-AGCT- 3’ as well as its iterations 5’-RGYW-3’ and 5’-WGCW (23) using DNA-Pattern at RSA tools (48). Raw counts were estimated in 2500 bp non-overlapping windows. As count distribution is Gaussian, window counts were transformed to Z-scores to allow comparability between different motifs.

### 4.6 Phylogenetic analysis of IGHV and IGLV segments

For the heavy chain *locus*, *H. sapiens* and *M. musculus* functional IGHV segments were retrieved from the IMGT (49). For *X. tropicalis* functional IGHV segments were retrieved from our own annotation. Multiple sequence alignment with the corresponding *A. mexicanum* IGHV segments was performed with MUSCLE (50). Average distance phylogenetic trees of the aligned sequences were done with JalView 2.10.5 (51) using a PAM250 scoring matrix.

For the comparative phylogenetic analysis of the IGL *locus*, the approach was identical as for IGHV, but using sequence data of **Supplementary File SD1.xls** from twelve tetrapods, in which the CDRL1 and CDRL2 were excluded. Positional numbering of cladistic markers was maintained accordingly (28).

### 4.7 RNA-seq analysis of immunoglobulin gene transcription

RNA seq data from different organs of project SRA: SRP101842 (spleen, liver, heart, lung, testes, ovary, brain, head, 9 and 15 day post - amputation forelimb blastemas, tail blastema, intact, 1 and 6 days post-injured spinal cord, limb and tail buds) were mapped also using STAR (46). Counts per gene were obtained with featureCounts v2.0.1 (52) using the mapping bam files. The relative frequencies of each gene were obtained using the total mapped sequences to all loci for each library as the denominator. To observe gene expression, heatmaps were built with the log10-transformed relative frequency of each gene using the ComplexHeatmap (53) library from R.

### 4.8 Intergenic and V-intron size analysis

A comparison of *A. mexicanum* V-V intergenic length was made with *D. rerio* (GRCz11) and *H. sapiens* (GRCh38.p12). In these cases, coordinate data was obtained from ENSEMBL gff dump (54). As for *X. tropicalis*, we performed a new *X. tropicalis* IGH *locus* annotation based on the corresponding ENSEMBL annotation (Xenopus_tropicalis_v9.1, GCA_000004195.3), further complemented and refined with sequence data kindly provided by Sibayshi Das (2), and *X. tropicalis* liver RNA-seq data (SRR579561) (55) following the same methodology as for *A. mexicanum*.

### 4.9 *A. mexicanum* IGH size ratio analysis

To calculate *A. mexicanum* IGH *locus*, IGHC cluster, IGHV cluster and individual C gene size ratios we measured the corresponding distance in the following ENSEMBL genome annotations (54): *D. rerio* (GRCz11), *H. sapiens* (GRCh38.p12), *M. musculus* (GRCm39), *Sarcophilus_harrisii* (mSarHar1.11), *Ornithorhynchus anatinus* (mOrnAna1.p.v1), *Anolis carolinensis* (AnoCar2.0v2), *Canis familiaris* (ROS_Cfam_1.0), *Gallus gallus* (bGalGal1.mat.broiler.GRCg7b), *Anas platyrhynchos* (ASM874695v1). Sizes of the bat *Rousettus agyptiacus* IGH locus were based on RaegypIGH3.0 (56). IGH *locus* length was defined from the start of the first IGHV gene to the end of last IGHC gene. IGHC *locus* was defined from the start of the first coding exon of IGHM to the end of the last exon of the most distal IGHC gene. Individual IGHC genes (from first to the last exon). To calculate IGHC ratios in mammals, the IGHA average length was used as IGHX ortholog and IGHG and IGHE average length was used as IGHY ortholog.

### 4.10 Intron length analysis

V-intron length was calculated from the exon coordinates of the respective *locus* annotation file. In the case of human and zebrafish, coordinate data were retrieved using BioMart from ENSEMBL (http://www.ensembl.org/biomart/martview/). Due to the presence of abnormally long introns (outliers) in *A. mexicanum*, a robust ANOVA One-Way Trimmed Means Comparison with a trimming level of 5% (tr = 0.05), followed by the *post hoc* Lincon test was performed (30). To calculate the enrichment of non-functional V genes according to intron length, a Fisher’s Exact test was implemented in R.

### 4.11 Whole-genome, IGHV, and *p450* intergenic length analysis

Amex_v6 gene annotation file, Biomart, Kruskal-Wallis test with *post hoc* Dunn test correction for multiple comparisons implemented in R.

## Data availability statement

The original contributions presented in the study are included in the article/**Supplementary Materials**. Further inquiries can be directed to the corresponding authors.

## Ethics statement

The animal study was reviewed and approved by Secretaría de Medio Ambiente y Recursos Naturales (SEMARNART) (SEMARNART: DGVS-PIMVS-CR-IN-1833/CDMX/17; N° SGPA/DGVS/04821).

## Author contributions

JMB conceived and designed the study and contributed to data analysis, interpretation and manuscript writing. CLM conceived, designed the study and manuscript writing. EGL contributed to data collection, analysis and interpretation. SSH and DPO contributed to data acquisition, analysis and drafting the manuscript. JMT and HVT contributed to Rep-seq data acquisition and analysis. LZ and HM provided and selected axolotl for the study, as well as training for animal handling, organ identification, and sample collection. RPP contributed to axolotl organ collection and samples processing. All authors contributed to the article and approved the submitted version.
