# Supplementary material for: Characterization of immunoglobulin loci in the gigantic genome of Ambystoma mexicanum
Source: Front Immunol. 2023 Jan 27;14:1039274. doi: 10.3389/fimmu.2023.1039274 (PMC9911811; doi:10.3389/fimmu.2023.1039274)
Supplement: Supplementary file 1 [file DataSheet_1.pdf]

Supplementary figures

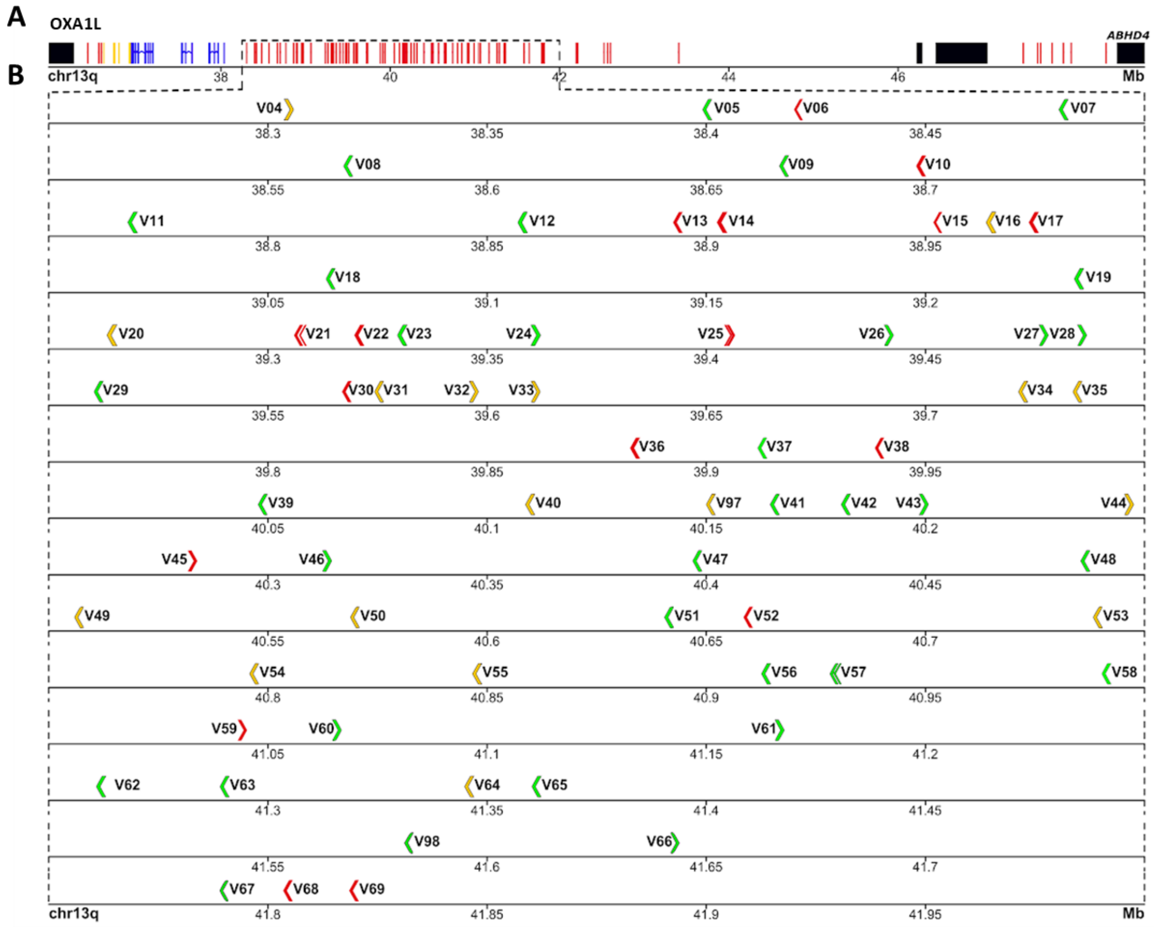

Supplementary Figure S1: Representation of the major IGHV cluster in *A. mexicanum* genome (v6) in chr13q. **A)** IGH *locus* (chr13q: 35.96 - 48.91 Mbp). Genes in black are non-Ig genes, IGHC in blue, IGHV genes in red, and IGHJ and IGHD genes in yellow. **B)** Zoomed region of the main IGHV gene cluster (chr13q: 38.25 - 42 Mbp). IGHV genes and their corresponding orientation are shown in arrows. Functional genes are in green, ORF's in yellow, and pseudogenes in red.

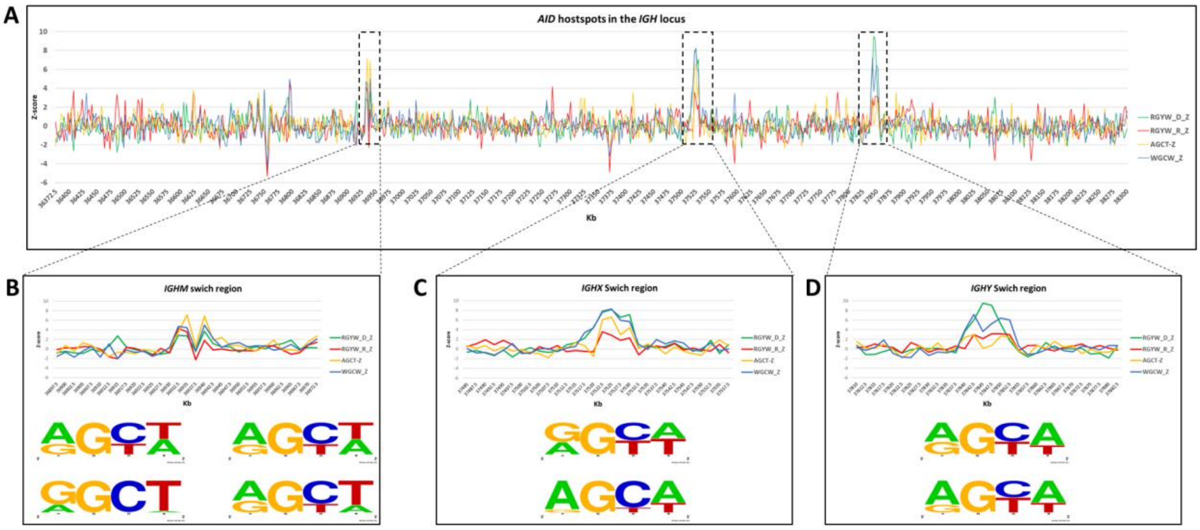

Supplementary Figure S2: Density of AID hotspot motifs along the IGHC cluster. The frequency of each motif distribution per 2.5 Kb was used to calculate the corresponding Z-score. The RGYW in the direct strand is shown in green. RGYW in the opposite strand is shown in red. The palindromic AGCT and WGCW motifs are shown in yellow and blue, respectively. **A)** Panoramic of the IGHC cluster (chr13q: 36,732.5-38,300 Kb). **B)** Close-up to the Sμ region. Note that the central region is depleted of RGYW motifs giving an "M" shape. The logos at the bottom represent frequency per site for each of two Sμ peaks. Top row (direct strand), bottom row (reverse strand). **C)** Close-up to the Sχ region and its corresponding logos (direct, Top; reverse, bottom). **D)** Close-up of the Sυ region and its corresponding motif logos (direct, top; reverse, bottom).

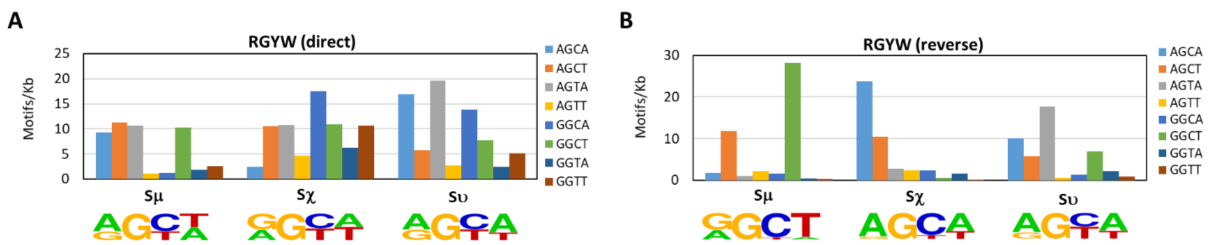

Supplementary Figure S3: Characterization of RGYW motif composition in the Sμ, Sχ and Sυ regions. The frequency per Kb of the 8 tetramers corresponding to the RGYW motif is shown per switch region. For Sμ, the RGYW-depleted central region (2.5Kb) was excluded from the computation. **A)** The direct strand, with the corresponding frequency per site plot; and **B)** The reverse strand, with the corresponding frequency per site plot.

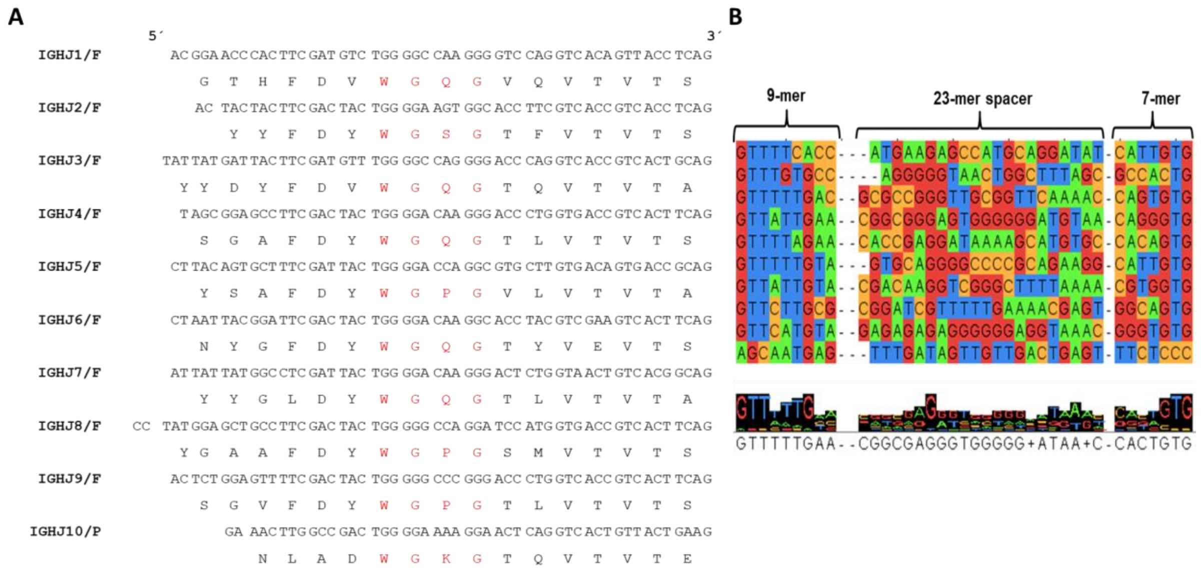

Supplementary Figure S4: Analysis of the structure of the *A. mexicanum* IGHJ segments. The Axolotl IGHJ cluster comprises ten mapped genes, of which nine are functional, and one is a pseudogene (IGHJ10). This cluster is located on chromosome 13q, upstream of the IGHC *locus*. **A)** Alignment of nucleotide and amino acid sequences of the ten identified JH segments, the conserved WGXG motif, characteristic of the IGH J-REGION, is in red. **B)** Nucleotide sequence alignment of the RSS (ordered according to A), showing the conserved 7 mer and 9 mer separated by a 23 bp spacer. Consider that the sequences shown correspond to the direct strand.

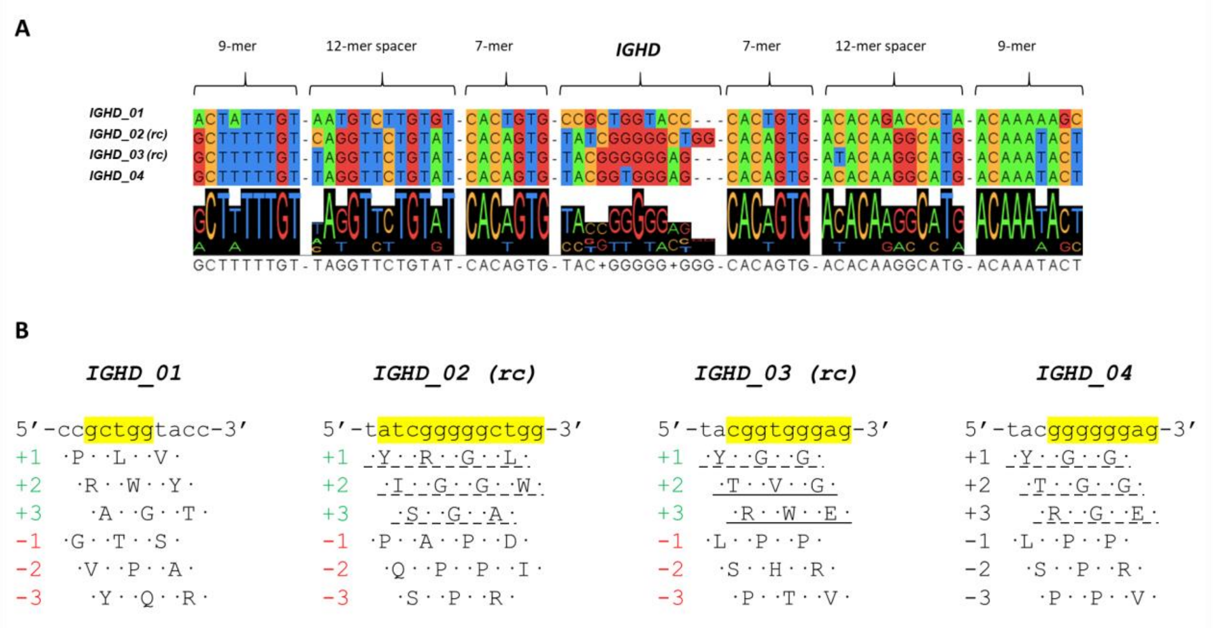

Supplementary Figure S5: Structural analysis of IGHD segments within the 36.6 - 36.8 Mbp interval in chr13q. **A)** Multiple alignments of the four identified IGHD plus the RSS's in both flanks show the conserved 9 mer and 7 mer separated by a 12 mer spacer on both flanks. Note that in the genome assembly, IGHD_02 and IGHD_03 are in reverse orientation so that the reverse complement (rc) is shown. **B)** Sequence translation of IGHD segments. The nucleotide sequence is shown in lower case. Identical positions to DH-like core sequences described by Golub, *et al*., 1997 are highlighted in yellow (IGHD_01 = DH1, IGHD_02 = DH4, IGHD_03 = DH2, IGHD_04 = DH3). Translation in six reading frames is shown in uppercase letters. Translation frames that are found in our spleen Rep-seq dataset are solid underlined, whereas translation frames shared in Golub et al. and our spleen Rep-seq dataset are dotted underlined. IGHD_01 translations were not found in either dataset. Note that although all translation frames are productive, only forward translations were found in the peripheral repertoire in both datasets.

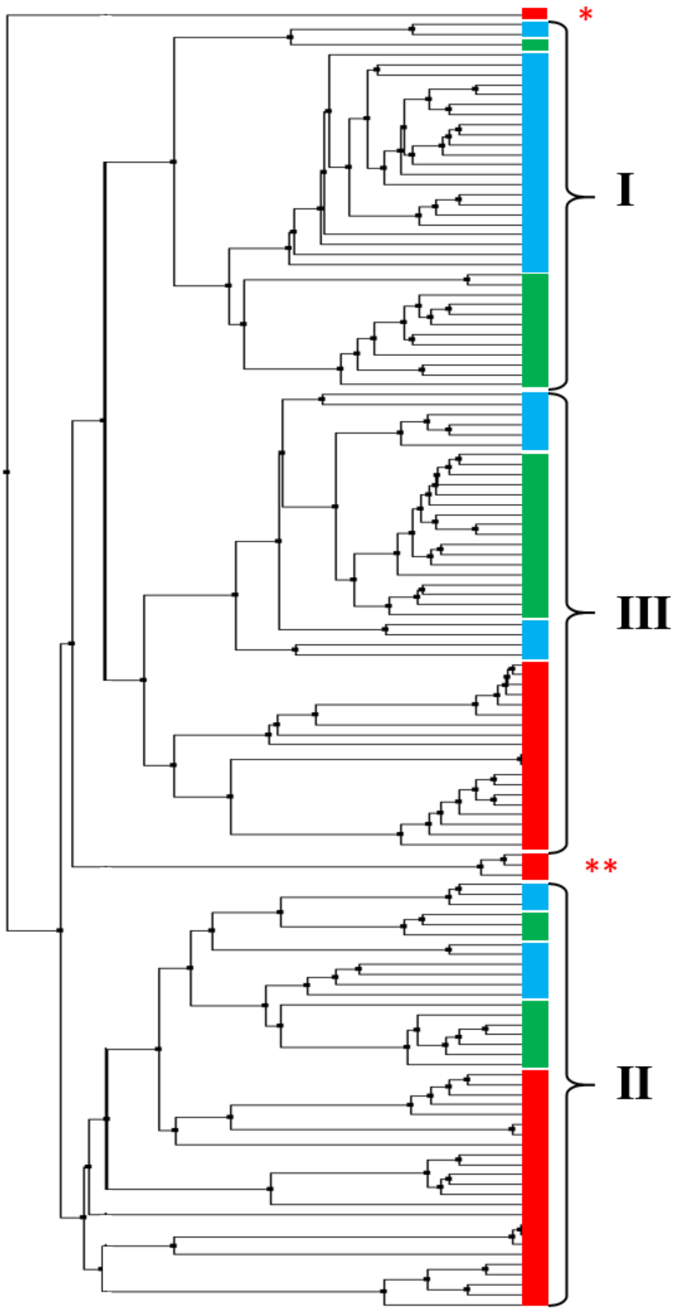

Supplementary Figure S6: Phylogenetic tree of human, mouse, and Axolotl functional IGHV genes. Representative Human (Green bar) and mouse (light blue bar) sequences for the three tetrapod IGHV clans were used as references to classify *A. mexicanum* IGHV sequences (Red bar). No clan-I IGHV sequences were found in *A. mexicanum*. Note that IGHV_082 is atypical and outroots the tree (*). Nevertheless, IGHV_082 qualifies as a functional gene because it contains a Variable Ig domain ORF including Cys23 and Cys104, putatively functional RSS, and transcribed. Another atypical case (**) corresponds to IGHV_70, 071, and 077, which out root from the clan I/III stem, so we could not assign them to either clan.

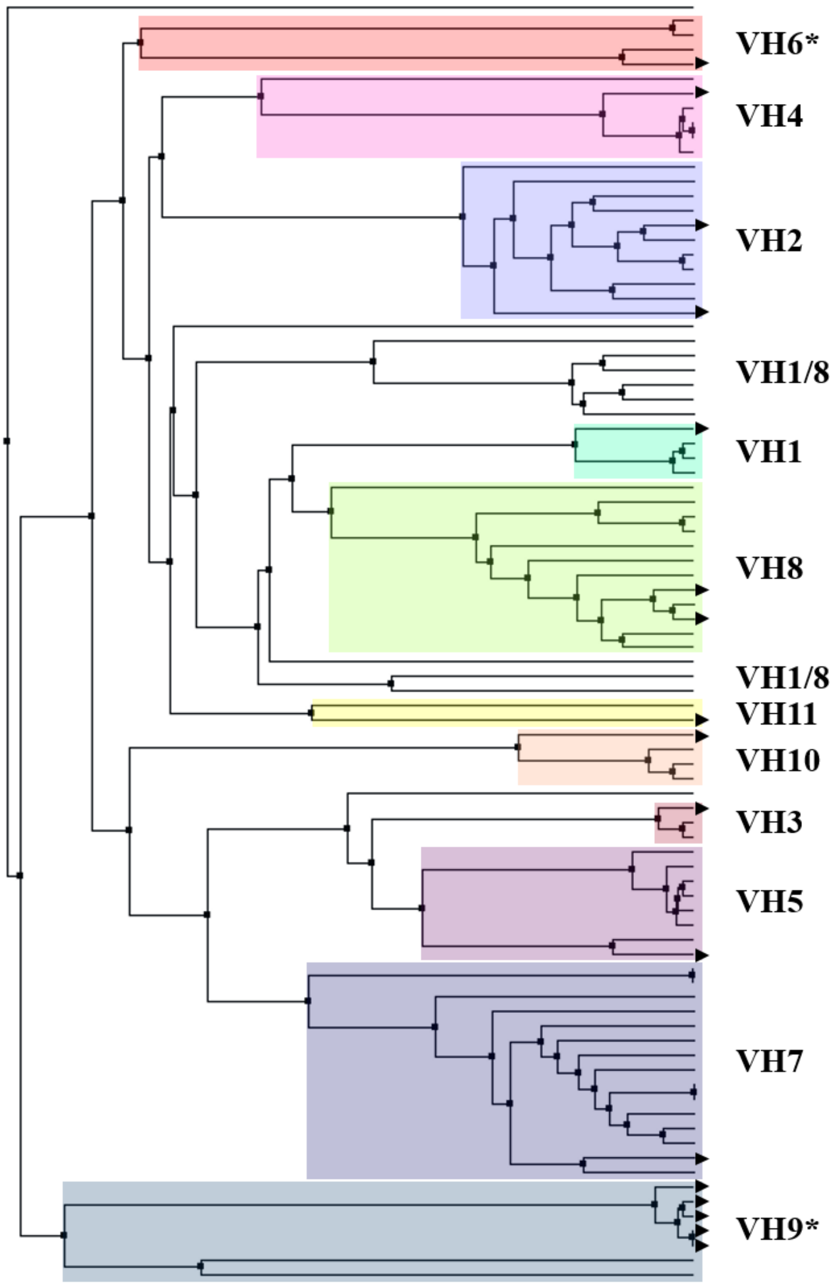

Supplementary Figure S7: IGHV families. Classification of *A. mexicanum* germline functional and ORF IGHV genes according to the 11 families described by Golub *et al*., 1998; based on axolotl VH cDNA analysis (black triangles). Germline sequences and representative cDNA sequences of each family used as reference were aligned with MUSCLE, and the phylogenetic tree was based on mean protein distance. No functional IGHV genes belonged to VH family VH6 and VH9 (*). Germline sequences with white background correspond to sequences that could not be assigned to a particular family.

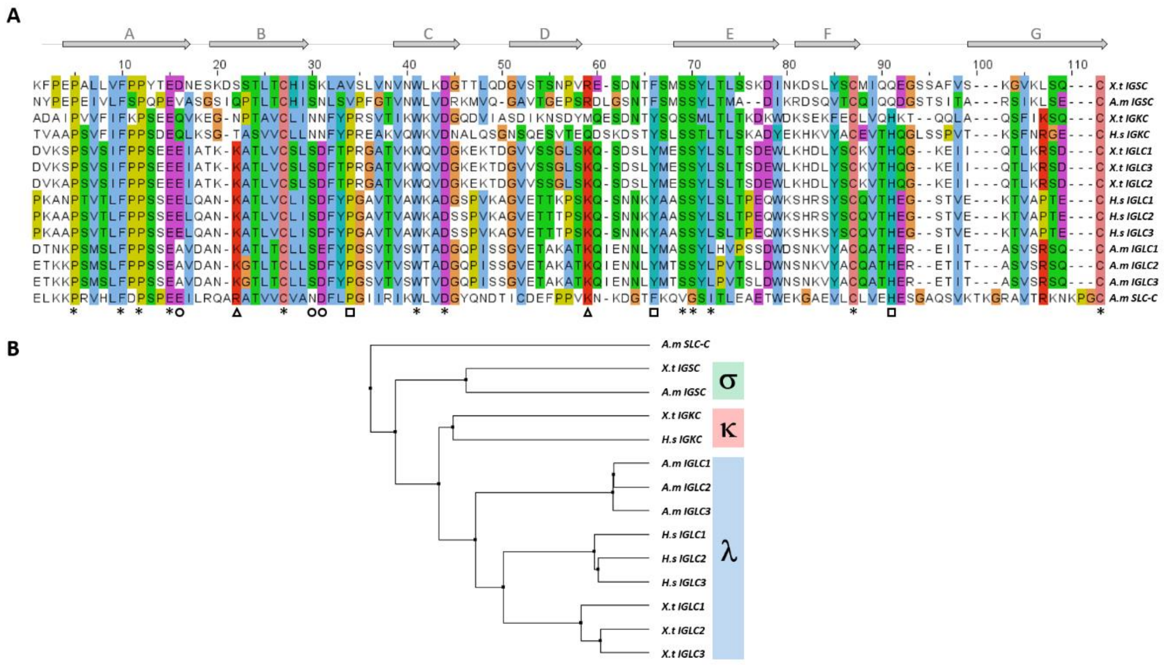

Supplementary Figure S8: Comparative analysis of the Constant Ig domain of light chains in humans and amphibians. A) Multiple alignments of Cλ in *A. mexicanum*, *X. tropicalis* and human (only 3 out of 5 functional Cλ), Cσ in *A. mexicanum* and *X. tropicalis,* Cκ in *X. tropicalis* and human, and the *A. mexicanum* putative surrogate light chain C-domain (SLC-C). The seven canonical β-sheets (A-G) are shown on top of the alignment. Asterisk shows twelve conserved residues in conventional light chains in the three species, including Cys28, Trp42, Cys87, and Cys104. Some of these residues are involved in contact with H chains, whereas Cys28 and Cys87 mediate intradomain disulfide bonding, and Cys104 forms a disulfide bond with the H chain. Triangles show Lys23 and Lys60, which are unique to Cλ. Squares indicate Pro33, Tyr65, and His90, which are conserved in Cλ and Cκ, but are replaced by Val, Phe, and Gln, respectively in Cσ. Circles show Gln17, Asp30, and Asp31 that are unique to Cκ. Residue numbering is based on André, *et al*., 2000. Alignment color is based on Clustal X coloring scheme. **B)** Average distance phylogenetic tree of Ig light chain C domain based on the multiple alignments shown in A. The C-type Ig domain of Cλ clusters together regardless of the species.

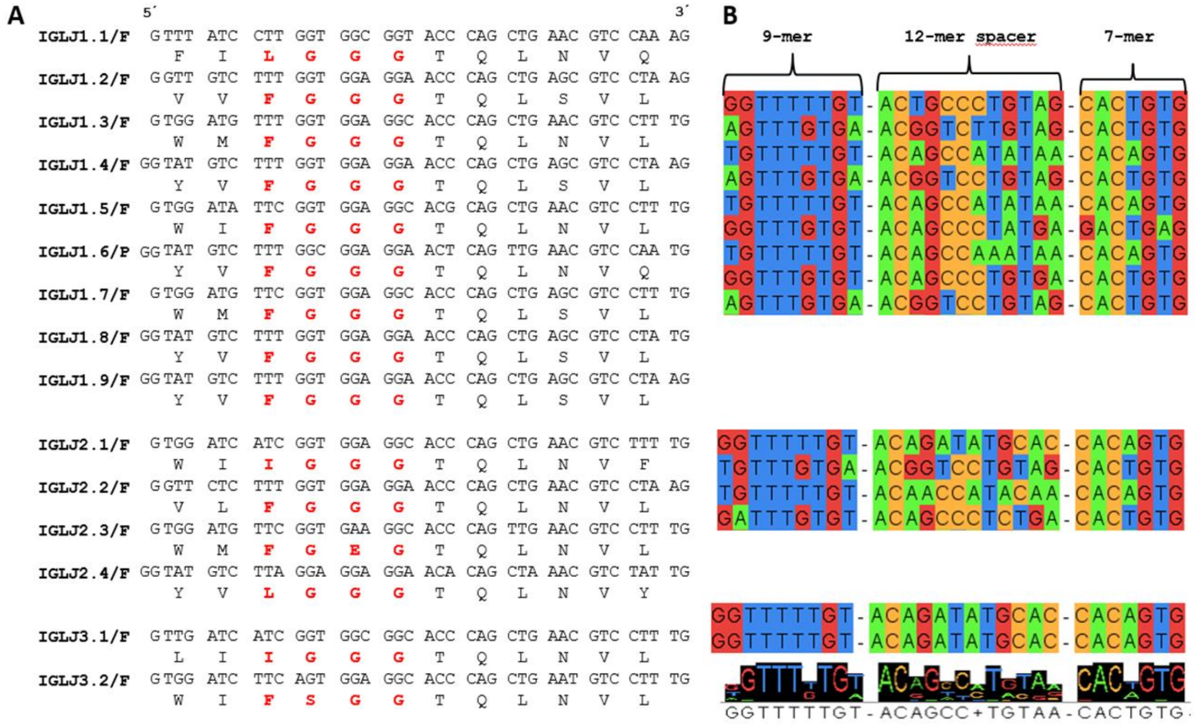

Supplementary Figure S9: Structural analysis of the *Ambystoma mexicanum* IGLJ segments. The Axolotl IGLJ group comprises three clusters. The first cluster includes nine mapped genes, of which eight are functional, and one is a pseudogene (IGLJ6). The second cluster comprises four mapped functional genes. These groups are located on chromosome 10q, flanked by the *DGCR2* gene on the centromeric flank and by the *CABIN1*. **A)** Alignment nucleotide and amino acid sequences of the ten identified IGLJ segments, the conserved FGXG motif (di-glycine bulk), characteristic of the IGL J-REGION, is in red. **B)** Alignment of nucleotide sequences of the RSS showing the conserved 7 mer and 9 mer separated by a 12 pb spacer. Considering that the sequences shown correspond to the direct chain 5'-3'.

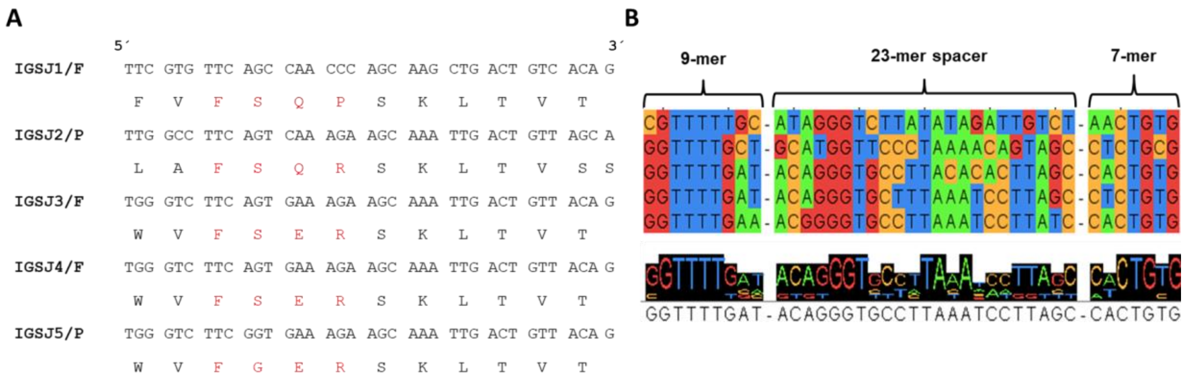

Supplementary Figure S10: Structural analysis of the *Ambystoma mexicanum* IGSJ segments. The axolotl IGSJ group comprises three functional segments and two pseudogenes (IGSJ2- IGSJ5). **A)** Nucleotide and amino acid sequence alignment of the five IGSJ segments, the conserved FSXR motif characteristic of the IGS J-REGION is in red. **B)** Alignment of nucleotide sequences of RSS showing the conserved 7 mer and 9 mer separated by a 23 pb spacer. Consider that the sequences shown correspond to the direct chain 5’-3'.

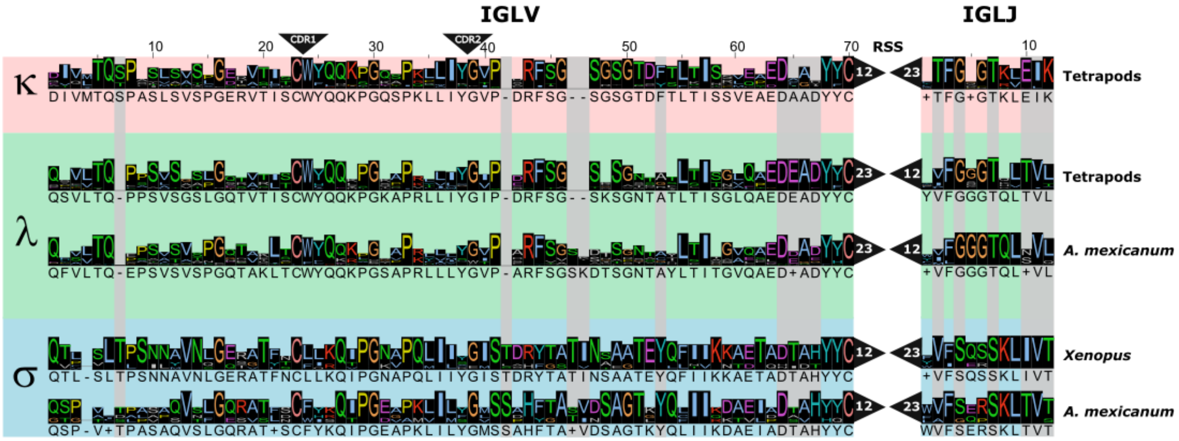

Supplementary Figure S11: Cladistic analysis of light chain *loci*. light chain protein sequences of tetrapods (V and J) were provided by S. Das (Das, *et al*., 2008), aligned with MUSCLE. Functional sequences of *A. mexicanum* were also included. The sequences corresponding to the CDRλ1 and 2 were removed. According to Das *et al*., 2008, cladistic markers are shown in gray. Note that the position of markers is according to Das *et al*., 2008 (without CDR1 and 2).

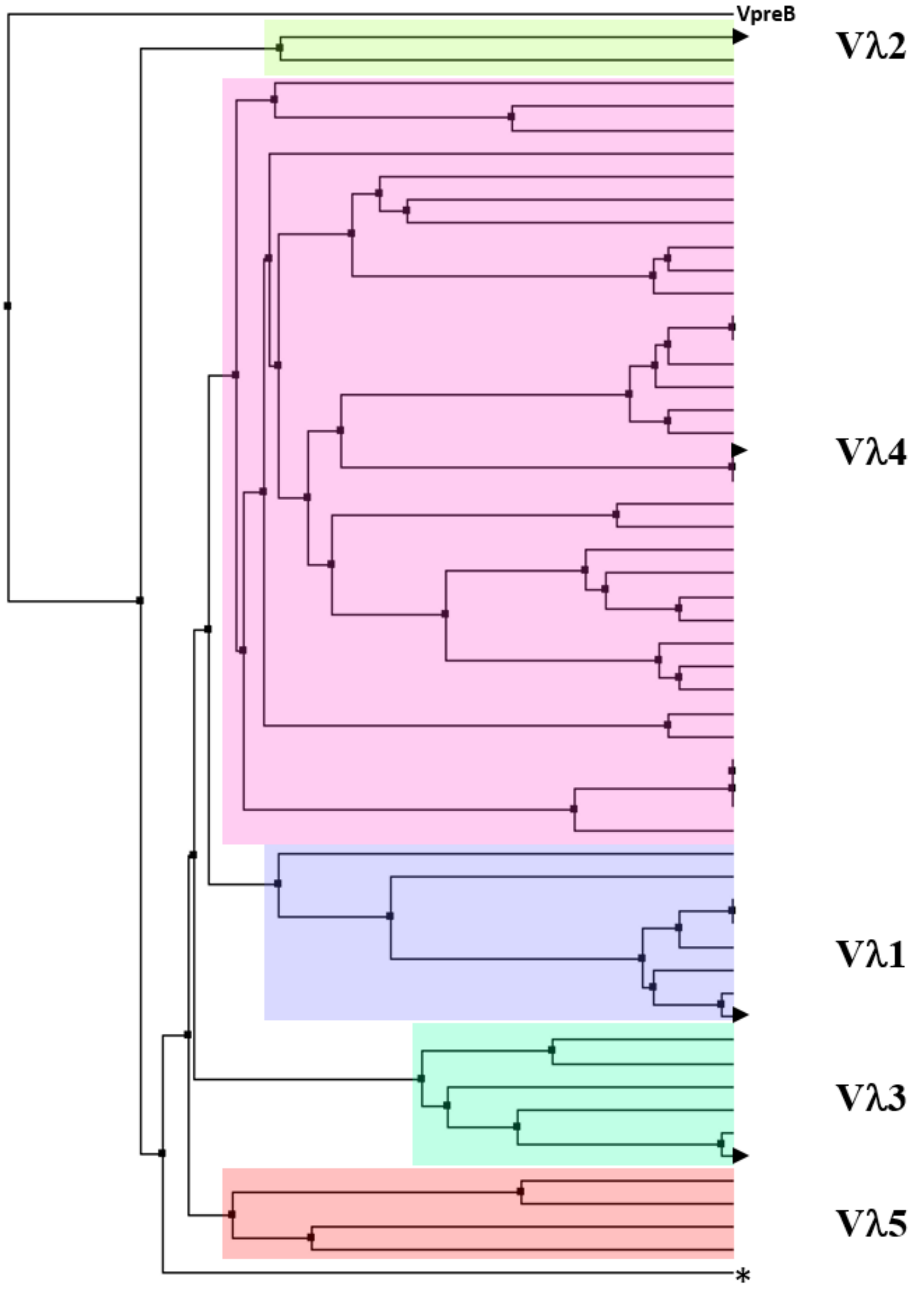

Supplementary Figure S12: Mean distance phylogenetic tree of *A. mexicanum* Vλ protein sequences. We used V1, V2, V3, and V4 sequences described by Golub as references (black triangles) to classify Vλ into previously described families (Accessions: AF317321.1, AF317322.1, AF317323.1, AF317324.1). The V domain of the putative SLC protein sequence was included to root the tree. We found functional Vλ genes in the four families, but Vλ4 was the most abundant (n = 29). Vλ2 had only one Vλ (IGLV_059). IGLV_040, IGLV_069, and IGLV_037 formed a previously unidentified family (Vλ5). IGLV_039 did not cluster with any family (*).

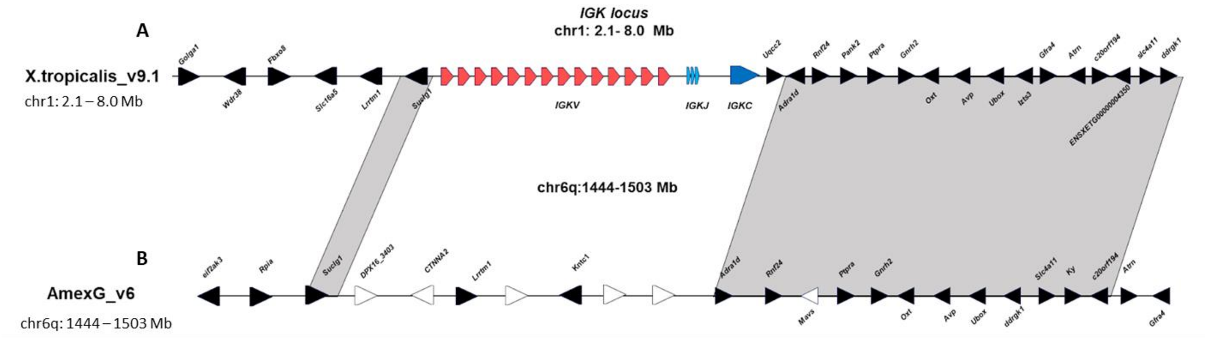

Supplementary Figure S13: Absence of the Ig kappa *locus* in the *A. mexicanum* genome (V6). Schematic representation of the **A)** *X. tropicalis* κ *locus* in chr1: 2.1 – 8.0 Mbp. The constant region gene is shown in dark blue, J cluster in light blue, and V cluster in red, regardless of their functionality. Non-Ig genes in black. *X. tropicalis* displays the canonical architecture, in which the V, J, and C clusters are in the same orientation. Note that in **B)** *A. mexicanum*, there is a complete absence of the kappa *locus*, and the downstream flank is located in chr6q: 1444 – 1503 Mbp. Two 1:1 orthologs in the upstream flank, *LRRTM1*, and *SUCLG1*, also map to the same region. Non-orthologous genes are shown as white-filled genes. Not on a scale.

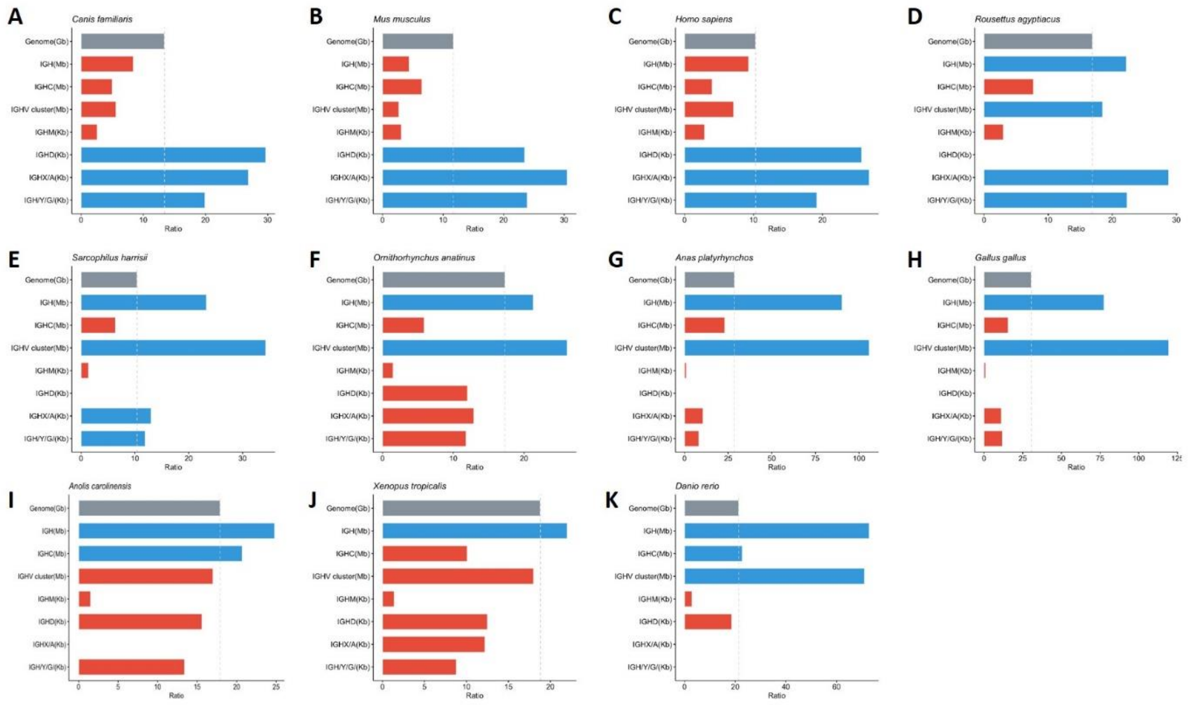

Supplementary Figure S14: IGHM gene length in *Ambystoma mexicanum* is evolutionarily constrained. *A. mexicanum* to genome, locus and ortholog size ratio comparison with placental mammals (A-D), non-placental mammals (E-F), birds (G-H), reptile (I), amphibian (J) and teleost fish (K). Genome size ratio is shown in grey bar, whereas ratios higher than the genome ratio are shown in blue bars, and size ratios below the genome ratio is shown in red bars. IGH *locus* length was defined from the first IGHV gene to the last exon of the last IGHC gene. IGHC *locus* was defined from the first coding exon of IGHM to last exon of the IGHC gene. Individual IGHC genes (From first to the last exon). To calculate IGHC ratios in mammals, the IGHA average length was used as IGHX ortholog and IGHG and IGHE average length was used as IGHY ortholog.

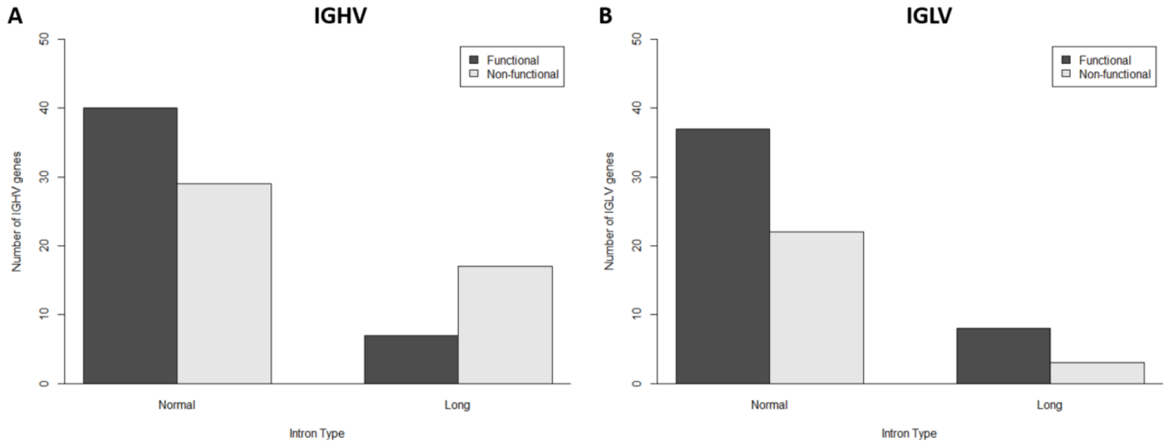

Supplementary Figure S15: Functionality according to the A) IGH and **B) IGL *loci* intron length.** A 2 x 2 contingency table was built with the number of functional and non-functional (pseudogenes plus ORF's) according to V-intron length (long V-intron > 150 bp) for IGHV (left) and IGLV (right). A Fisher's exact test revealed that the odds of an IGHV gene with a long V-intron being non-functional are 3.3 higher than its short V-intron counterpart (P = 0.018, CI95: 1.1, 10.7). In contrast, for the IGLV *locus*, there were no differences in the observed and expected frequencies of short and long V-introns according to functionality (P = 0.73; OR = 0.63, CI95: 0.09, 3.0).
